# Curcumin β-D-Glucuronide Modulates an Autoimmune Model of Multiple Sclerosis with Altered Gut Microbiota in the Ileum and Feces

**DOI:** 10.3389/fcimb.2021.772962

**Published:** 2021-12-03

**Authors:** Sundar Khadka, Seiichi Omura, Fumitaka Sato, Kazuto Nishio, Hideaki Kakeya, Ikuo Tsunoda

**Affiliations:** ^1^ Department of Microbiology, Kindai University Faculty of Medicine, Osaka, Japan; ^2^ Department of Genome Biology, Kindai University Faculty of Medicine, Osaka, Japan; ^3^ Graduate School of Pharmaceutical Sciences, Kyoto University, Kyoto, Japan

**Keywords:** bioinformatics, animal model, pattern matching, PICRUSt analysis, bacterial taxonomy, Alpha diversity, confidence interval, histology

## Abstract

We developed a prodrug type of curcumin, curcumin monoglucuronide (CMG), whose intravenous/intraperitoneal injection achieves a high serum concentration of free-form curcumin. Although curcumin has been reported to alter the gut microbiota and immune responses, it is unclear whether the altered microbiota could be associated with inflammation in immune-mediated diseases, such as multiple sclerosis (MS). We aimed to determine whether CMG administration could affect the gut microbiota at three anatomical sites (feces, ileal contents, and the ileal mucosa), leading to suppression of inflammation in the central nervous system (CNS) in an autoimmune model for MS, experimental autoimmune encephalomyelitis (EAE). We injected EAE mice with CMG, harvested the brains and spinal cords for histological analyses, and conducted microbiome analyses using 16S rRNA sequencing. CMG administration modulated EAE clinically and histologically, and altered overall microbiota compositions in feces and ileal contents, but not the ileal mucosa. Principal component analysis (PCA) of the microbiome showed that principal component (PC) 1 values in ileal contents, but not in feces, correlated with the clinical and histological EAE scores. On the other hand, when we analyzed the individual bacteria of the microbiota, the EAE scores correlated with significant increases in the relative abundance of two bacterial species at each anatomical site: *Ruminococcus bromii* and *Blautia (Ruminococcus) gnavus* in feces, *Turicibacter* sp. and *Alistipes finegoldii* in ileal contents, and *Burkholderia* spp. and *Azoarcus* spp. in the ileal mucosa. Therefore, CMG administration could alter the gut microbiota at the three different sites differentially in not only the overall gut microbiome compositions but also the abundance of individual bacteria, each of which was associated with modulation of neuroinflammation.

## 1 Introduction

Polyphenol curcumin is the principal active component of turmeric, *Curcuma longa* (Ozawa et al., 2017). Experimentally, curcumin has been reported to have multiple functions, including antioxidant (Lin et al., 2019), antitumor (Ozawa et al., 2017), and anti-inflammatory functions (Xie et al., 2009); curcumin has been shown to be beneficial in several disease conditions including multiple sclerosis (MS) (Mohajeri et al., 2015), cancer (De Velasco et al., 2020), and inflammation (Xie et al., 2009). Clinically, however, the oral administration of free-form curcumin did not provide the desired effects in clinical trials. This can be explained by its rapid metabolism in the body; curcumin is metabolized to an inactive conjugated form after it is taken from the intestine (Asai and Miyazawa, 2000), although only a free-form of curcumin is associated with the pharmacological activity. In addition, the bioavailability of curcumin within the body is inadequate due to the poor absorption from the intestine, insolubility in body fluids, and rapid elimination and clearance from the body through feces (Anand et al., 2007; Ozawa et al., 2017).

Since the low bioavailability of curcumin in the body has limited its application as a therapeutic agent, we developed a prodrug type of curcumin, curcumin β-D-monoglucuronide (curcumin monoglucuronide, CMG). CMG is safe and can be injected intravenously; CMG administration achieved a high concentration of free-form curcumin in the blood of rats (Ozawa et al., 2017). We demonstrated that intravenous CMG administration had an anticancer effect on mice transplanted with human colorectal cancer cells by achieving the blood concentration of free-form curcumin 1000-fold more than oral administration of curcumin (Ozawa et al., 2017). Intraperitoneal CMG administration also had anti-tumor effects on oxaliplatin-resistant colon cancer with less toxicity in mouse xenograft models (Ozawa-Umeta et al., 2020).

Abundant and diverse microbial communities coexist in mammals, including humans and mice. In the gastrointestinal (GI) tract, the microbial communities are composed of microorganisms, including bacteria and archaea, which are collectively referred as the gut “microbiota”. Recently, the communications between the gut microbiota and immune system have been shown to contribute to eliminating microbes and cancers by activating systemic immune responses (Lazar et al., 2018). In contrast, dysbiosis, an altered state of microbiota, has been shown to induce uncontrolled excessive immune responses (Park et al., 2017; Gandy et al., 2019), leading to immune-mediated tissue damage, immunopathology, not only in the GI tract but also in other organs, including the central nervous system (CNS) (Braniste et al., 2014). Although curcumin has been reported to potentially alter both the gut microbiota and immune responses, it is unknown which gut microbial communities can be affected by curcumin and whether the altered microbiota could be associated with suppression of immunopathology in immune-mediated diseases, such as MS.

We aimed to determine whether CMG administration could affect the gut microbiota at three anatomical sites: feces, ileal contents, and the ileal mucosa. We also investigated that the altered microbiota by CMG administration could be associated with suppression of immunopathology, using an autoimmune model for MS (Chearwae and Bright, 2008), experimental autoimmune encephalomyelitis (EAE). In MS, the gut microbiota has been proposed to play a role in disease progression and severity. Clinically, MS patients have had increased abundance of some bacteria, including the genera *Akkermansia*, *Pseudomonas*, and *Blautia*, and decreased abundance of some bacteria, such as the genera *Prevotella* and *Parabacteroides*, compared with the healthy controls (Miyake et al., 2015; Chen et al., 2016; Jangi et al., 2016; Park et al., 2017; Tsunoda, 2017). EAE can be induced by sensitizing animals with myelin components, such as myelin oligodendrocyte glycoprotein (MOG) and myelin proteolipid protein (PLP). Anti-myelin autoimmune cells cause inflammatory demyelination in the CNS, resulting in paralysis in EAE animals, which resembles MS (Sato et al., 2018). Pro-inflammatory T helper (Th)1/Th17 cytokines, including interferon (IFN)-γ and interleukin (IL)-17, contribute to the development of EAE; anti-inflammatory Th2/regulatory T (Treg) cytokines, including IL-4 and IL-10, are protective in EAE (Xie et al., 2009; Chaudhry et al., 2011).

Although curcumin has been reported to alter the gut microbiota of rodents and humans (Di Meo et al., 2019; Zam, 2018; Peterson et al., 2018) in healthy and disease conditions, including EAE (Shen et al., 2017; Zhang et al., 2017), the effect of curcumin on the gut microbiota at different anatomical sites and their associations of disease conditions have not been clarified. In the present study, we demonstrated that CMG administration modulated EAE, where the severity was associated with altered overall microbiota composition in ileal contents, but not in feces or the ileal mucosa. On the other hand, when we analyzed the relative abundance of individual bacteria at the three anatomical sites, the EAE severities also correlated with significant increases in the relative abundance of two bacterial species at each anatomical site: *Ruminococcus bromii* and *Blautia (Ruminococcus) gnavus* in feces, *Turicibacter* sp. and *Alistipes finegoldii* in ileal contents, and *Burkholderia* spp. and *Azoarcus* spp. in the ileal mucosa. Therefore, CMG administration could differentially alter the gut microbiota at the three different sites in not only overall gut microbiome compositions, but also the abundance of individual bacterial species, each of which was associated with decreased inflammation in the CNS.

## 2 Materials and Methods

### 2.1 Mice

Six-week-old female C57BL/6 mice were purchased from CLEA Japan, Inc. (Tokyo, Japan). The mice were maintained under specific-pathogen-free conditions in our animal care facility at Kindai University Faculty of Medicine (Osaka, Japan). All experimental procedures were approved by the Institutional Animal Care and Use Committee of Kindai University Faculty of Medicine and performed according to the criteria outlined by the National Institutes of Health (National Research Council (US) Committee for the Update of the Guide for the Care and Use of Laboratory Animals, 2011).

### 2.2 EAE Induction and CMG Administration

For EAE induction, mice were sensitized subcutaneously (s.c.) with 258 μg (=100 nmol) of the MOG_35-55_ peptide (MEVGWYRSPFSRVVHLYRNGK, United BioSystems, Herndon, VA) emulsified in complete Freund’s adjuvant (CFA) that is composed of incomplete Freund’s adjuvant (BD, Franklin Lakes, NJ) and *Mycobacterium tuberculosis* H37 Ra (BD) on days 0 and 19 (Fernando et al., 2014). The final concentration of *M. tuberculosis* in the MOG_35-55_/CFA emulsion was 2 mg/mL (400 μg/mouse). The mice were also injected intraperitoneally (i.p.) with 300 ng of pertussis toxin (List Biological Laboratories, Campbell, CA) on days 0 and 2. The clinical scores of EAE were evaluated as follows: 0, no sign; 1, tail paralysis; 2, mild hindlimb paresis; 3, moderate hindlimb paralysis; 4, complete hindlimb paraplegia; and 5, quadriplegia or moribund state (Tsunoda et al., 2007). The cumulative scores were calculated by the area under the EAE score curve that reflects the overall disease severity over the course.

A CMG solution was prepared in phosphate-buffered saline (PBS) at a concentration of 9 mg/mL and stored at ‒80°C until used (Ozawa et al., 2017; Ozawa-Umeta et al., 2020). The mice were divided into four groups (7‒9 mice per group): the Control, Induction, Latent, and Whole groups based on the CMG administration schedule. The mice were injected i.p. with 200 µL of the CMG solution (1.8 mg/mouse) on days 0‒4 (Induction group), on days 11‒15 (Latent group), or on days 0‒4, 6, 8, 9, 11‒15, 17, 19, 21, 23, 25, 27, 29, 31, and 33 (Whole group). In the Whole group, the mice were treated daily with CMG on days 0–4 and 11–15 using the same administration schedules as the Induction and Latent groups, respectively, and three times a week in the other time points, based on our previous publications (Ozawa et al., 2017; Ozawa-Umeta et al., 2020). The control mice (Control group) were injected with PBS. To determine the effects of CMG administration on EAE mice, their body weight changes and EAE scores were monitored daily for 5 weeks.

### 2.3 Neuropathology

The mice were killed with isoflurane (Mylan N.V., Canonsburg, PA) 5 weeks post-induction (p.i.) and perfused with PBS followed by a 4% paraformaldehyde (PFA, FUJIFILM Wako Pure Chemical Corporation, Osaka, Japan) solution in PBS (Sato et al., 2017). After the PFA fixation, the spinal cord and brain were divided into 12 to 15 transversal segments and five coronal slabs, respectively, and were embedded in paraffin. Four-μm-thick CNS sections were made using the HM 325 Rotary Microtome (Thermo Fisher Scientific Inc., Waltham, MA) and stained with Luxol fast blue (Solvent Blue 38; MP Biomedicals, LLC, Irvine, CA) for myelin visualization. The phenotypes of immune cells were determined by immunohistochemistry. The CNS sections were incubated with antibodies against CD3 (T cell marker, 100-fold dilution, Biocare Medical, Pacheco, CA; antigen retrieval: 10 mM citrate buffer pH 6.0 at 120°C for 15 min), B220 (B cell marker, 300-fold dilution, eBioscience, San Diego, CA; no antigen retrieval), F4/80 (macrophage marker, 200-fold dilution, Bio-Rad, Hercules, CA; antigen retrieval: 100 µg/ml proteinase-K for 10 min), Ly-6G (neutrophil marker, 500-fold dilution, BD Bioscience, Franklin Lakes, NJ; no antigen retrieval), Foxp3 (Treg marker, 100-fold dilution, eBioscience; antigen retrieval: 10 mM citrate buffer pH 6.0 at 120°C for 15 min), and goat anti-mouse IgA-UNLB antibody (IgA-producing cell marker, 2000-fold dilution, SouthernBiotech, Birmingham, AL; no antigen retrieval) (Omura et al., 2020). The antibody/antigen complexes were visualized using 3,3’-diaminobenzidine (DAB, FUJIFILM Wako Pure Chemical Corporation).

### 2.4 Enzyme-Linked Immunosorbent Assays (ELISAs)

When the mice were killed 5 weeks p.i., the spleens and inguinal lymph nodes were harvested and mashed on a metal mesh with 50-μm pores. Splenic mononuclear cells (MNCs) were isolated using Histopaque^®^-1083 (MilliporeSigma, Burlington, MA). The splenic MNCs and lymph node cells were cultured in RPMI-1640 medium (MilliporeSigma) supplemented with 10% fetal bovine serum (FBS, MilliporeSigma), 2 mM L-glutamine (MilliporeSigma), 50 mM β-mercaptoethanol (FUJIFILM Wako Pure Chemical Corporation), and a 1% antibiotics solution (Thermo Fisher Scientific, Waltham, MA) containing 10,000 U/mL penicillin and 10,000 μg/mL streptomycin at concertation of 8 × 10^6^ cells/well in 6-well plates (Sumitomo Bakelite, Tokyo, Japan) (Martinez et al., 2014). The cells were stimulated with 5 µg/mL of the mitogen concanavalin A (ConA, MilliporeSigma) or 50 µg/mL of the MOG_35−55_ peptide for 2 days. The culture supernatants were harvested and stored at ‒80°C until examined.

The amounts of IL-4 (BD Biosciences, San Jose, CA), IL-10 (BD Biosciences), IFN-γ (BD Biosciences), and IL-17A (Biolegend, San Diego, CA) in the culture supernatants were quantified using ELISA kits, according to the manufacturers’ instructions (Tsunoda et al., 2005). The detection limits of each cytokine were as follows: IL-4, 7.8 pg/mL; IL-10, 31.3 pg/mL; IFN-γ, 31.3 pg/mL; and IL-17A, 15.6 pg/mL. ELISAs were conducted in duplicate, using 96-well plates (Thermo Fisher Scientific).

### 2.5 Gut Microbiota Sample Collection

After perfusing mice with PBS, for microbiome analyses, the fecal samples (also called “feces” in this manuscript) were collected 5 weeks p.i. from the rectum and/or the anal canal, and the ileal content samples were harvested from the ileum by flushing with distilled water. The flushed ileum was rinsed with Dulbecco’s Modified Eagle’s Medium (DMEM, MilliporeSigma, Burlington, MA) containing 10% fetal bovine serum (FBS) twice and incubated in DMEM containing 10% FBS and 1 mM dithiothreitol (DTT, MilliporeSigma) with shaking for 40 min. The supernatants were filtered with Falcon^®^ 70-μm cell strainers (Corning Incorporated, Corning, NY) and centrifuged at 5,000*g* for 15 min at 4°C. After centrifugation, the pellets were used as the samples containing the gut microbiota from the ileal mucosa. All samples were frozen in liquid nitrogen and stored at –80°C until examined (Tong et al., 2014).

### 2.6 DNA Extraction and Sequencing

Bacterial DNA was extracted from feces, ileal contents, and the ileal mucosa using the QIAamp^®^ Fast DNA Stool Mini Kit (Qiagen, Germantown, MD), according to the manufacturer’s instructions (Omura et al., 2020). Using the DNA samples, 16S rRNA amplicon sequencing was conducted on MiSeq System (Illumina, San Diego, CA) by MR DNA (Shallowater, TX), a commercially available sequencing service, who processed the sequence data using a MR DNA analysis pipeline (http://www.mrdnalab.com/bioinformatics.html). Primer sequences used for sequencing (515F/806R) were designed against the V4 region of the 16S rRNA as follows: forward (515F), 5’-AATGATACGGCGACCACCGAGATCTACACTATGGTAATTGTGTGCCAGCMGCCGCGGTAA-3’; reverse (806R), 5’-CAAGCAGAAGACGGCATACGAGATCTAGCGTGCGTTAGTCAGTCAGCCGGACTACHVGGGTWTCTAAT-3’ (Caporaso et al., 2011). Operational taxonomic units (OTUs) were defined by clustering at 3% divergence (97% similarity). The final OTUs were classified taxonomically using BLASTn against a curated database derived from the Ribosomal Database Project (RDP)-II (http://rdp.cme.msu.edu) and National Center for Biotechnology Information (NCBI, www.ncbi.nlm.nih.gov). Total read count number in our analysis ranged from 22,488 to 29,674 reads (average: 29,245 reads); the number of read counts was similar among the three anatomical sites. The sequencing depth was within this range in all samples. To determine the diversities and conduct PICRUSt (phylogenetic investigation of communities by reconstruction of unobserved states) metagenome prediction analyses, the raw sequence data were denoised, demultiplexed, aligned, and visualized by QIIME 2 (Bolyen et al., 2019). The data generated and analyzed in the current study have been deposited into the Sequence Read Archive (SRA) at NCBI (BioProject ID: PRJNA688384, BioSample accessions: SAMN17174018-SAMN17174101).

### 2.7 Bioinformatics Analyses

#### 2.7.1 Alpha Diversity

The alpha diversity of microbiomes at the genus level were compared among the three distinct anatomical sites and among the four groups at each anatomical site using QIIME 2 (Bolyen et al., 2019). To determine the bacterial richness, evenness, and combination of richness/evenness, the Faith’s phylogenetic diversity, Pielou’s evenness, and Shannon indexes were calculated, respectively (Omura et al., 2020).

#### 2.7.2 Principal Component Analysis (PCA)

To compare the overall microbiomes among all samples from the three distinct anatomical sites and among individual samples from each anatomical site, PCA was conducted using an R program “prcomp”, as described previously (Chaitanya et al., 2013). Factor loading for principal component (PC) was used to rank individual bacteria whose relative abundance was correlated with PC values. A graph of PCA with ellipses of an 80% confidence interval was drawn, using R packages “dplyr” and “ggplot2”.

#### 2.7.3 Pattern Matching

To examine the associations between the EAE severity and gut microbiota, pattern matching was conducted by R, using the clinical and neuropathological scores of EAE versus PC1 values of the microbiome at each anatomical site or versus the relative abundance of individual bacteria at the species level (Omura et al., 2019). The values more than 0.7 or less than ‒0.7 in the Spearman’s rank correlation coefficient (*r*) with *P* < 0.05 (calculated by functions of Microsoft Excel, Microsoft Corporation, Redmond, WA) were considered as a highly positive or negative correlation, respectively (Mukaka, 2012). The *r* value, which is from 0.5 to 0.7 or from ‒0.7 to ‒0.5 with *P* < 0.05, was considered as a moderate correlation.

#### 2.7.4 Predictive Metagenome Analysis

The functional composition of a metagenome of the microbiota was predicted by running PICRUSt on the Linux operating system, as described previously (Omura et al., 2020). By analyzing 16S rRNA sequencing data of the gut microbiota by PICRUSt (Langille et al., 2013), the following two data files were obtained: 1) a pathway-based prediction data file containing lists of a) the name of pathways, b) the total read count data of all bacteria encoding genes related to each pathway, and c) KEGG pathway names; and 2) an enzyme-based metagenome prediction data file containing lists of a) the enzyme ID number, b) the total read count data of all bacteria encoding the enzyme, and c) KEGG enzyme names. Using the pathway prediction data, the read counts were compared between the Control and CMG-treated groups, and the pathways with significant differences (*P* < 0.05, compared with the Control group, Student’s *t* test) were identified. Using the metagenome prediction data, the read counts of all bacteria encoding β-glucuronidase were also compared between the Control and CMG-treated groups.

### 2.8 Statistical Analyses

Using the OriginPro 2020 (OriginLab Corporation, Northampton, MA), the Mann–Whitney *U* test and Student’s *t* test and ANOVA with Fisher’s PLSD test as *post hoc* test were conducted for nonparametric and parametric data, respectively.

## 3 Results

### 3.1 CMG Administration Modulates EAE

**Figure 1:**
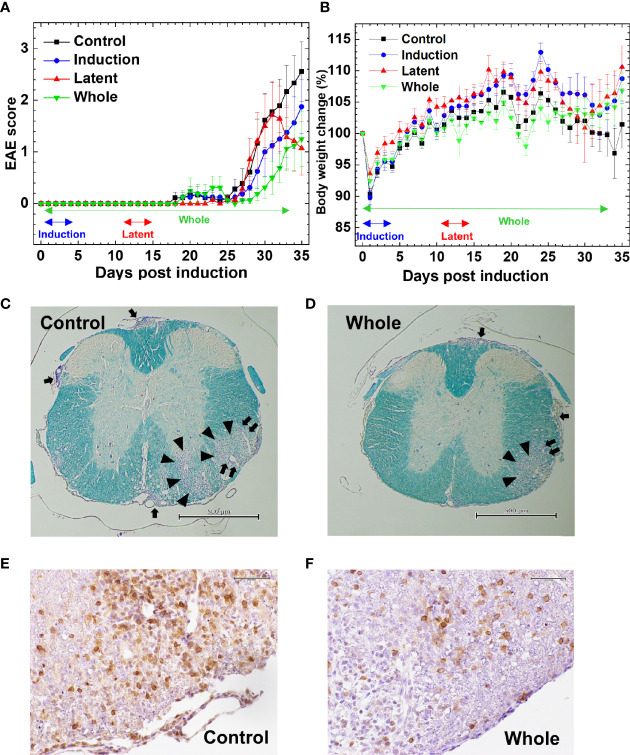
Effects of curcumin monoglucuronide (CMG) in an autoimmune model of multiple sclerosis (MS), experimental autoimmune encephalomyelitis (EAE). **(A)** For EAE induction, we sensitized C57BL/6 mice with the myelin oligodendrocyte glycoprotein (MOG)_35-55_ peptides. We divided the mice into four groups: Control, Induction, Latent, and Whole groups (n = 7–9 per group), and treated the groups with CMG on days 0–4 (Induction), on days 11–15 (Latent), or throughout the course (Whole). We treated the control mice (Control) with phosphate-buffered saline (PBS). All CMG-treated groups had less severe EAE compared with the control group. The Whole group tended to have a lower EAE score than the Control group 30 days post-induction (p.i.) (*P* = 0.06, Mann–Whitney *U* test). The Latent group tended to recover from acute EAE more quickly than the Control group 35 days p.i. (*P* = 0.056, Mann–Whitney *U* test). The clinical score was calculated as the mean ± standard error (SE) of seven to nine mice per group. **(B)** We monitored body weight changes of mice daily. The levels of body weight changes had an inverse relationship to the severity of clinical signs. The Control EAE group had more weight loss compared with the Induction (*P* = 0.06), Latent (*P* = 0.07), and Whole (*P* = 0.14) groups on day 34. Results are the mean ± SE of seven to nine mice per group. **(C–F)** We stained the spinal cord sections (scale bar: C and D = 500 µm, E and F = 50 µm) from EAE mice with Luxol fast blue **(C, D)** or anti-CD3 antibody **(E, F)** to visualize myelin or T cells, respectively, compared the spinal cord pathology between the Control **(C, E)** and CMG-treated Whole groups **(D, F)**. The Whole group had less severe demyelination (arrowhead), meningitis (arrow), and perivascular inflammation (paired arrows) and fewer T cell infiltration, compared with the Control group. The stained sections are representative of seven to nine mice per group.

We tested whether CMG administration could affect EAE. Following MOG sensitization, we treated mice with CMG during the induction (Induction group) or latent phase (Latent group), or throughout the whole course (Whole group). We injected the control EAE mice (Control group) with PBS. We monitored the EAE signs (**Figure 1A**) and body weight changes (reflecting the severity of EAE, **Figure 1B**) daily for 5 weeks. All CMG-treated groups (Induction, Latent, and Whole) tended to develop less severe EAE compared with the Control group; less weight loss in the Induction and Latent group (**Figure 1B**) and lower cumulative scores of EAE in the Whole group (**Supplemental Table 1A**). Although CMG-treated groups had lower maximum clinical scores, EAE incidence, and EAE duration compared with the Control group, these parameters did not reach statistical differences (**Supplemental Table 1A**).

The severity of EAE correlated with neuropathology; mice from the Latent and Whole groups developed less severe inflammatory demyelinating lesions in the spinal cord (**Figures 1C–F**). Although the neuropathology scores did not reach statistical differences, there were some trends toward lower levels of perivascular inflammation (cuffing) and demyelination in the spinal cord in the Latent and Whole groups compared with the Control group (**Supplemental Figure 1A**). On the other hand, there were no differences in the spinal cord pathology between the Control and Induction groups. We also compared the brain pathology scores among the four groups and found no differences [mean brain pathology scores ± standard error (SE) 5 weeks p.i.: Control, 3.9 ± 0.7; Induction, 4.1 ± 0.9; Latent, 3.6 ± 1.8; Whole, 3.1 ± 0.4]. To further determine the effects of CMG on neuropathology, we conducted hematoxylin and eosin staining to see whether neutrophil infiltration differed among the groups. We found that CNS cellular infiltrates were mainly composed of mononuclear cells (MNCs), but not neutrophils, in all groups (**Supplemental Figure 1B**); a small number of neutrophil infiltration was confirmed by immunohistochemistry using Ly-6G antibody (**Supplemental Table 1B** and **Supplemental Figure 1E**). Next, we compared the phenotypes of MNCs in the spinal cord by immunohistochemistry using antibodies against CD3 (T cell marker), B220 (B cell marker), and F4/80 (macrophage marker) (**Figures 1E, F**, **Supplemental Figures 1C, D** and **Supplemental Table 1B**). The percentages of CD3^+^ T cells in CMG-treated groups were lower than the control group: Control, 57.6%; Induction, 52%; Latent, 46%; and Whole, 33%) (**Supplemental Table 1B**). Although we detected a small number of B cells in inflammatory areas and a large number of F4/80 positive macrophage lineage cells in inflammatory areas and demyelinating lesions, B-cell and macrophage infiltrations were similar among the groups (**Supplemental Table 1B**). Since regulatory T (Treg) cells and IgA-producing B cells have been shown to regulate MS and animal models, we compared Treg cells and IgA^+^ cells among the groups using Foxp3 and IgA immunohistochemistry (**Supplemental Figures 1F, G**). We found similar levels of Foxp3^+^ T cells among the groups. We did not find IgA^+^ cells in any group in the spinal cord, although IgA was detected in the control intestine sections.

We cultured splenic MNCs and lymph node cells from EAE mice and quantified the amounts of IL-10, IL-4, IFN-γ, and IL-17 in the cultures stimulated with mitogen or MOG by ELISAs. Although splenic MNCs of the Latent group tended to produce larger amounts of IL-10 in mitogen stimulation and IFN-γ in MOG stimulation compared with controls (*P* < 0.1), there were no statistical differences in the cytokine levels among the four groups in the other cultures (**Supplemental Figure 2**). We also examined the levels of MOG-specific lymphoproliferation and found that all groups had substantial MOG-specific lymphoproliferative responses without significant differences among the four groups (**Supplemental Figure 3**).

### 3.2 Microbiota in EAE Differs Among the Three Anatomical Sites

Since the microbiota has been reported to be different between the luminal contents versus mucosa in the same anatomical site as well as the distinct anatomical sites in humans and mice (Li et al., 2015; Wu et al., 2020), we harvested the microbial samples from feces, ileal contents, and the ileal mucosa of EAE mice. Using 16S rRNA sequencing data, we determined bacterial alpha diversities by the Faith’s phylogenetic diversity (species richness), Pielou’s evenness (species evenness), and Shannon (combination of richness and evenness) indexes. As expected, the three indexes using the all samples were significantly different among the three anatomical sites (*P* < 0.05, ANOVA, **Supplemental Figure 4**). We also found that the overall microbiome patterns were different between the samples from feces, ileal contents, and the ileal mucosa by PCA of the microbiome data (**Supplemental Figure 5**). PCA separated the ileal mucosal samples from the fecal and ileal content samples by PC1 at all three taxonomical levels: phylum, genus, and species (**Supplemental Figure 6**). PC1 values were significantly higher in the ileal mucosa than in feces and ileal contents at the three taxonomical levels (*P* < 0.05, ANOVA). PC2 values were also significantly different among the three anatomical sites at the genus and species levels, but not the phylum level (**Supplemental Figure 7**).

### 3.3 CMG Administration Alters the Fecal and Ileal Microbiota

**Figure 2:**
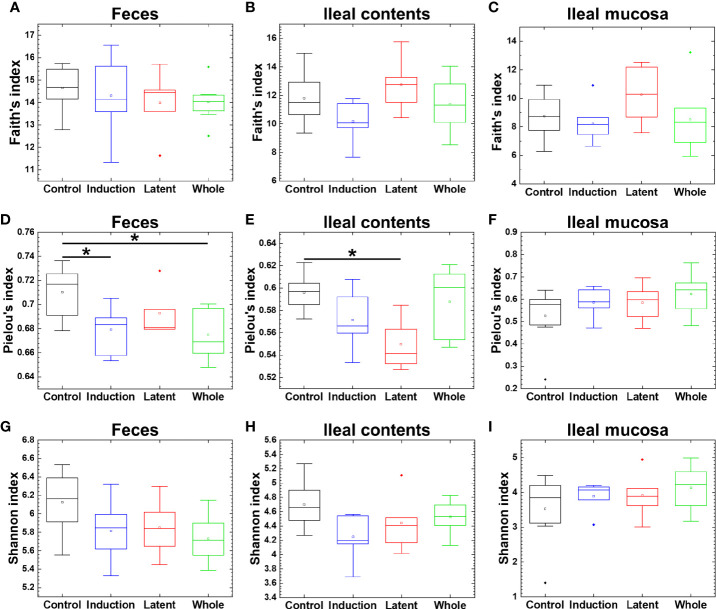
Analyses of bacterial alpha diversities of the microbiome at three anatomical sites from EAE mice with or without CMG administration. Using QIIME 2, we compared the number of genera, evenness, and combination of them by the Faith’s phylogenetic diversity **(A–C)**, Pielou’s evenness **(D–F)**, and Shannon **(G–I)** indexes, respectively, between the three CMG administration (Induction, blue; Latent, red; and Whole, green) and control groups (Control, black). We found no difference in the Faith’s **(A–C)** or Shannon index **(G–I)** among the CMG-treated and Control groups, although the Pielou’s index was significantly different between the Control versus Whole, the Control versus Induction groups in feces **(D),** and the Control versus Latent groups in ileal contents **(E)**, all of which had decreased diversity in the CMG-treated groups (*P < 0.05, Kruskal-Wallis test) **(D, E)**.

Since a reduced microbial diversity has been associated with some diseases, including inflammatory bowel diseases (IBDs) (Manichanh et al., 2006; Gong et al., 2016), we determined whether CMG administration could alter the diversity of the microbiota at each anatomical site. Using the three indexes, however, we found that the microbial diversity in the CMG-treated groups was similar to that in the Control group (**Figure 2**). There were no differences in the Faith’s or Shannon index among the four groups. The Pielou’s index was similar in the ileal mucosa between the Control and CMG-treated groups, although it was higher in the Control group compared with the Whole and Induction groups in feces and the Latent groups in ileal contents.

**Figure 3:**
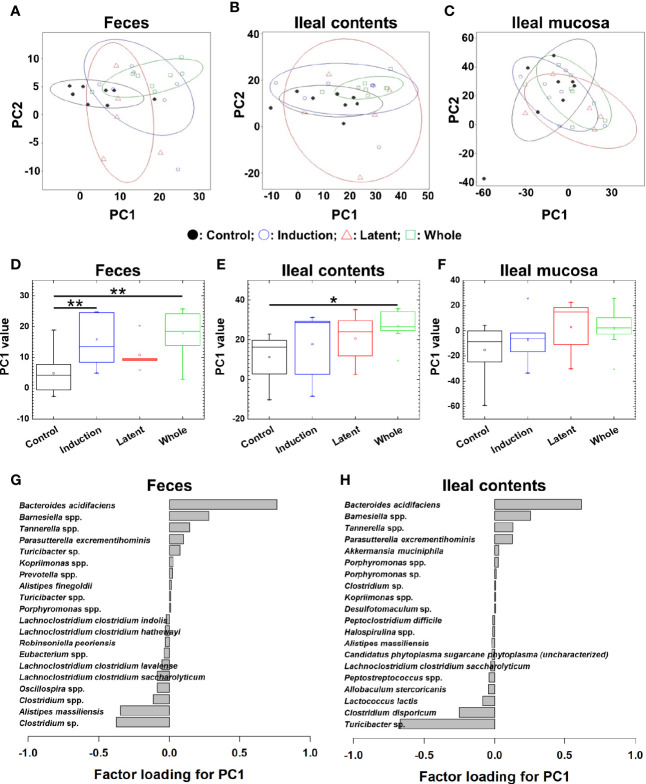
Principal component analysis (PCA) of microbiome data at the species level from the three CMG-treated groups (Induction, blue; Latent, red; and Whole, green) and the control group (Control, black). **(A–C)** We conducted PCA using samples from the three anatomical sites: feces **(A)**, ileal contents **(B)**, and the ileal mucosa **(C)**. Ellipses indicated an 80% confidence interval of each group. Proportion of variance of principal component (PC)1 and PC2 were 47% and 14% in feces, 52% and 27% in ileal contents, and 43% and 37% in the ileal mucosa. **(D)** In feces, PC1 values were significantly different between the Control versus Induction and the Control versus Whole groups (**P < 0.01, ANOVA). **(G)** Factor loading for PC1 showed that the relative abundance of the species *Bacteroides acidifaciens* most highly correlated with PC1 values. **(E)** In ileal contents, PC1 values were significantly different between the Control versus Whole groups (*P < 0.05, ANOVA). **(H)** Factor loading for PC1 showed that the relative abundance of *Bacteriodes acidifaciens* and *Turcibacter* sp. correlated positively and negatively with PC1 values, respectively. **(F)** In the ileal mucosa, PC1 values were not different among the groups.

We also tested whether CMG administration could alter the overall microbiota compositions in feces, ileal contents, and the ileal mucosa at the phylum, genus, and species levels. At the three taxonomical levels, PCA of the microbiota data from feces and ileal contents, but not the ileal mucosa, separated the CMG-treated groups from the Control group by PC1 (**Figures 3A–C** and **Supplemental Figures 8A–C**, **9A–C**). PC1 values were significantly higher in the feces and ileal contents of all CMG-treated groups than those of the Control group (**Figures 3D–F** and **Supplemental Figures 8D–F**, **9D–F**). Factor loading for PC1 indicated that, at the species level, increased *Bacteroides acidifaciens* and decreased *Clostridium* sp. and *Alistipes massiliensis* in feces as well as increased *Bacteroides acidifaciens* and decreased *Turcibacter* sp. in ileal contents correlated with PC1 values (**Figures 3G, H**). These results demonstrated that CMG administration affected microbiota in feces and ileal contents, but not in the ileal mucosa.

### 3.4 CMG Administration Alters the Bacterial Abundance at the Three Anatomical Sites

**Figure 4:**
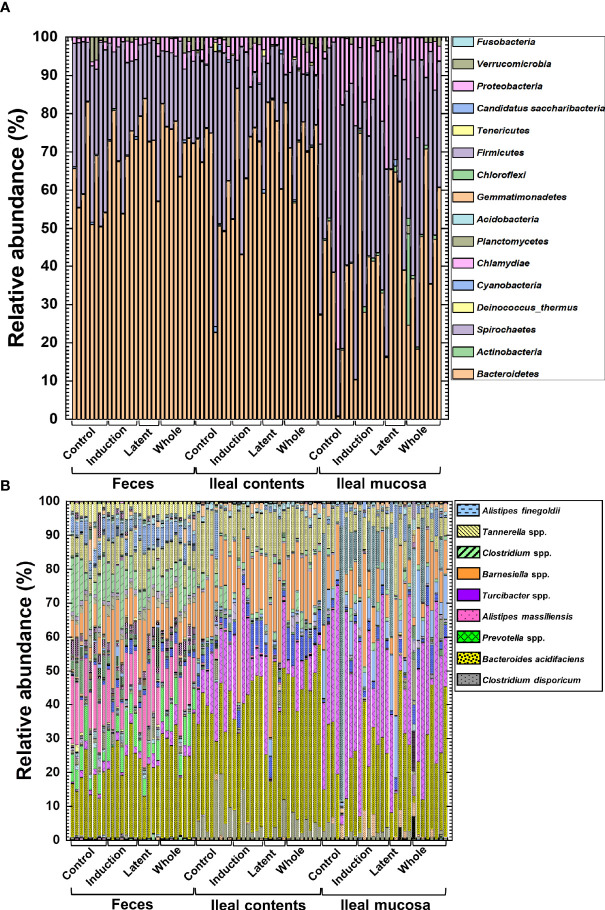
The relative abundance of bacteria from the three anatomical sites: feces, ileal contents, and the ileal mucosa. **(A, B)** Using 16S rRNA sequencing, we analyzed the relative abundance of individual bacteria at the phylum **(A)** and species **(B)** levels. We harvested samples from the three CMG-treated (Induction, Latent, and Whole) and control groups (Control). At both phylum and species levels, compositional differences in the microbiota between the three anatomical sites were larger than the microbiota differences among the CMG-treated and control groups. Each group was composed of five to eight mice.

We prepared naïve, CFA/PT-injected, and EAE mice as the control groups. However, since the relative abundance of naïve and CFA/PT mice was much different from the four EAE groups (**Supplemental Figure 10**), we decided to evaluate the microbiota changes by CMG treatment by comparing the three CMG-treated groups with the control EAE group, but not with the naïve or CFA/PT group (**Figure 4**). We compared the relative abundance of individual bacteria among feces, ileal contents, and the ileal mucosa at the phylum, genus, and species levels (**Figure 4**, **Supplemental Figure 11**, and **Supplemental Tables 2**, **3**). We found significant differences in the bacterial abundance among the three anatomical sites: 7 of total 16 taxa at the phylum levels, 78 of total 252 taxa at the genus levels, and 160 of total 387 taxa at the species level (**Supplemental Table 2**).

Then, we examined whether CMG administration could affect the relative abundance of individual bacteria. We found significant compositional differences of the microbiota between the Control versus CMG-treated groups at the phylum, genus, and species levels (**Figure 4** and **Tables 1**–**3**). At the phylum and genus levels, CMG administration altered the largest numbers of bacterial taxa in feces and the fewest numbers of bacterial taxa in the ileal mucosa. Among the CMG-treated groups, the Whole group had the largest numbers of altered relative abundance of bacteria compared with the Control group. The changes of the microbiota seemed to depend on the timing of CMG administration; although the numbers of bacteria with altered relative abundance were similar between the Induction and Latent groups, the altered bacterial phyla and genera were different between the two groups. For example, at the phylum level, the decreased abundance of *Firmicutes* in feces and increased abundance of *Proteobacteria* in ileal contents were seen in the Induction and Whole groups, but not in the Latent group, in which the decreased abundance of *Cyanobacteria* was seen (**Table 1**).

**Table 1:** Bacterial phyla altered in the curcumin monoglucuronide (CMG)-treated groups compared with the Control group.

Groups	Feces	Ileal contents	Ileal mucosa
**Induction**	*Firmicutes* ***↓***	*Proteobacteria* ***↑***	(–)
**Latent**	*Cyanobacteria* ***↓***	(–)	(–)
**Whole**	*Firmicutes* ***↓***	*Proteobacteria* ***↑***	(–)
	*Bacteroidetes* ***↑***	*Firmicutes* ***↓***	
	*Proteobacteria* ***↑***		

↑, Significant increases compared with the Control group (P < 0.05, ANOVA).

↓, Significant decrease compared with the Control group (P < 0.05, ANOVA).

(–), No differences compared with the Control group.

**Table 2:** Bacterial genera altered in the CMG-treated groups compared with the Control group.

Groups	Feces		Ileal contents		Ileal mucosa	
	↑	↓	↑	↓	↑	↓
**Induction**	*Bacteroides* *Enterorhabdus* *Parasutterella*	*Butyricicoccus Syntrophococcus Marvinbryantia*	*Parasutterella*	*Coprococcus* *Subdoligranulum*	(–)	(–)
**Latent**	*Enterorhabdus* *Pseudobutyrivibrio* *Bacillus*	*Roseburia* *Ruminiclostridium* *Clostridium* *Alloprevotella* *Gloeobacter*	*Senegalimassilia*	*Parabacteroides*	*Blautia*	(–)
**Whole**	*Bacteroides* *Parasutterella* *Olsenella* *Kopriimonas* *Turicibacter*	*Dehalobacterium* *Roseburia* *Ruminococcus* *Clostridium* *Lachnoclostridium* *Alloprevotella* *Erysipelatoclostridium* *Butyricicoccus* *Marvinbryantia* *Syntrophococcus* *Ruminiclostridium* *Anaerosporobacter* *Fastidiosipila*	*Parasutterella* *Azoarcus* *Olsenella* *Bacteroides*	*Odoribacter* *Syntrophococcus* *Parabacteroides* *Turicibacter*	*Parasutterella*	*Enterorhabdus* *Azoarcus* *Alistipes*

↑, Significant increases compared with the Control group (P < 0.05, ANOVA).

↓, Significant decrease compared with the Control group (P < 0.05, ANOVA).

(–), No differences compared with the Control group.

**Table 3:** Numbers of bacterial species altered in the CMG-treated groups compared with the Control group.

Samples	Feces		Ileal contents		Ileal mucosa	
	↑	↓	↑	↓	↑	↓
**Induction**	5^a^	7^b^	*Barnesiella* sp.*, Parasutterella excrementihominis*	6^c^	*Bifidobacterium choerinum*	3^d^
**Latent**	*Enterorhabdus mucosicola, Pseudobutyrivibrio* spp.	5^e^	*Senegalimassilia anaerobia*	*Parabacteroides distasonis*, *Alistipes putredinis*	*Blautia* sp.	0
**Whole**	4^f^	16^g^	6^h^	8^i^	*Parasutterella excrementihominis*	8^j^

↑, increased bacterial species; ↓, decreased bacterial species.

**A. Feces**

a Induction↑: Pseudobutyrivibrio spp., Enterorhabdus mucosicola, Parasutterella excrementihominis, Barnesiella sp., Bacteroides acidifaciens.

b Induction↓: Pseudoflavonifractor bacteroides capillosus, Butyricicoccus pullicaecorum, Lachnoclostridium clostridium hathewayi, Lachnoclostridium clostridium aldenense, Marvinbryantia bryantella formatexigens, Blautia (Ruminococcus) gnavus, Syntrophococcus sp.

e Latent↓: Alloprevotella rava, Gloeobacter spp., Blautia (Ruminococcus) gnavus, Ruminiclostridium eubacterium siraeum, Roseburia faecis.

f Whole↑: Olsenella spp., Parasutterella excrementihominis, Kopriimonas spp., Bacteroides acidifaciens.

g Whole↓: Alloprevotella rava, Dehalobacterium spp., Pseudoflavonifractor bacteroides capillosus, Butyricicoccus pullicaecorum, Anaerosporobacter mobilis, Clostridium spp., Roseburia faecis, Lachnoclostridium clostridium hathewayi, Ruminococcus sp., Eubacterium plexicaudatum, Marvinbryantia bryantella formatexigens, Fastidiosipila sanguinis, Ruminococcus bromii, Blautia (Ruminococcus) gnavus, Syntrophococcus sp., Erysipelatoclostridium clostridium innocuum.

Induction↑ and Latent↑: Enterohabdus mucosicola, Pseudobutyrivibrio spp. (Increases in Enterorhabdus mucosicola and Pseudobutyrivibrio spp. in the Induction and Latent groups, but not in the Whole group).

Induction↓ and Whole↓: Marvinbryantia bryantella formatexigens, Syntrophococcus sp. (Marvinbryantia bryantella formatexigens and Syntrophococcus sp. decreased in the Induction and Whole groups).

Latent↓ and Whole↓: Roseburia faecis (The Latent and Whole groups had a decrease in Roseburia faecis).

Induction↓, Latent↓, and Whole↓: Blautia (Ruminococcus) gnavus [All CMG-treated groups had a decrease in Blautia (Ruminococcus) gnavus].

**B. Ileal contents**

c Induction↓: Clostridium spp., Ruminiclostridium eubacterium siraeum, Blautia (Ruminococcus) gnavus, Blautia sp., Subdoligranulum spp., Coprococcus catus.

h Whole↑: Azoarcus spp., Pandoraea sp., Olsenella spp., Eubacterium xylanophilum, Parasutterella excrementihominis, Bacteroides acidifaciens.

i Whole↓: Turicibacter sp., Parabacteroides distasonis, Alistipes finegoldii, Alistipes putredinis, Odoribacter splanchnicus, Lachnoclostridium clostridium polysaccharolyticum, Clostridium fusiformis, Syntrophococcus sp.

Induction↑ and Whole↑: Parasutterella excrementihominis.

Latent↓ and Whole↓: Paracteroides distansonis, Alistipes putredinis.

**C. Ileal mucosa**

d Induction↓: Lachnoclostridium clostridium saccharolyticum, Lachnoclostridium clostridium hathewayi, Clostridium perfringens.

j Whole↓: Clostridium disporicum, Alistipes massiliensis, Azoarcus spp., Lachnoclostridium clostridium saccharolyticum, Enterorhabdus mucosicola, Burkholderia spp., Clostridium fusiformis, Clostridium perfringens.

Induction↓ and Whole↓: Lachnoclostridium clostridium saccharolyticum, Clostridium perfringes.

Similarly, at the species level, CMG administration resulted in the largest and fewest alterations of the microbiota in feces and the ileal mucosa, respectively; the three CMG-treated groups had common alterations of the relative abundance in limited numbers of bacterial species (**Figure 4B** and **Table 3**). For example, in feces, we found significant changes in 12 species in the Induction group, seven species in the Latent group, and 20 species in the Whole group, among which the relative abundance of *Blautia (Ruminococcus) gnavus* was decreased in all three CMG-treated groups compared with the Control group.

### 3.5 Ileal Content Microbiota Associates With Clinical and Histological EAE

**Figure 5:**
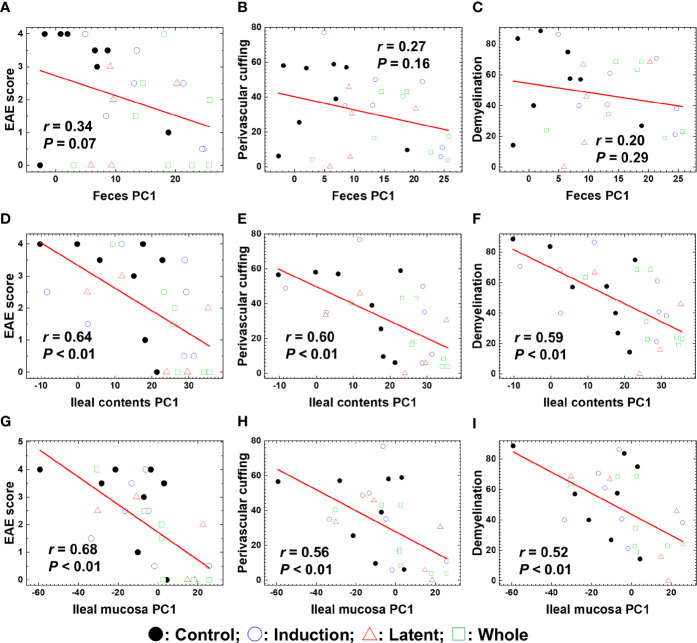
Pattern matching of the microbial PC1 values with the clinical and pathological scores of EAE at the three anatomical sites: feces, ileal contents, and the ileal mucosa. **(A–I)** We harvested samples from the three CMG-treated (Induction, blue circle; Latent, red triangle; and Whole, green square) and control groups (Control, black circle). The microbial PC1 values of ileal contents and the ileal mucosa, but not feces, significantly correlated with the EAE scores **(A, D, G)**, perivascular cuffing (i.e., inflammation) scores **(B, E, H)**, and demyelination scores **(C, F, I)** (*P* < 0.01).

To determine whether the overall changes in the microbiota by CMG administration could correlate with the severity of EAE, we conducted pattern matching between the clinical and neuropathology scores of EAE versus PC1 values of microbiota data at the species level (**Figure 5**). Although PC1 values of the fecal samples and EAE scores were not correlated (**Figure 5A**), PC1 values of ileal contents and the ileal mucosa correlated moderately with the EAE scores (*P* < 0.01, **Figures 5D, G**). Similarly, there were moderate correlations between PC1 values of ileal contents/mucosa versus the neuropathology scores including inflammation (i.e., perivascular cuffing) and demyelination with statistical differences (*P* < 0.01, **Figures 5E, F, H, I**); we found no correlations between fecal PC1 values versus the neuropathology scores (**Figures 5B, C**). Thus, bacterial PC1 values of ileal contents, but not feces, had significant correlations with the severity of EAE, although CMG administration changed PC1 values of the microbiota in feces and ileal contents (**Figure 3**). This suggests that CMG administration may have beneficial effects in EAE by altering the microbiota in ileal contents. On the other hand, in general, the microbiota in the ileal mucosa may be associated with the EAE severity irrespective of CMG administration.

**Figure 6:**
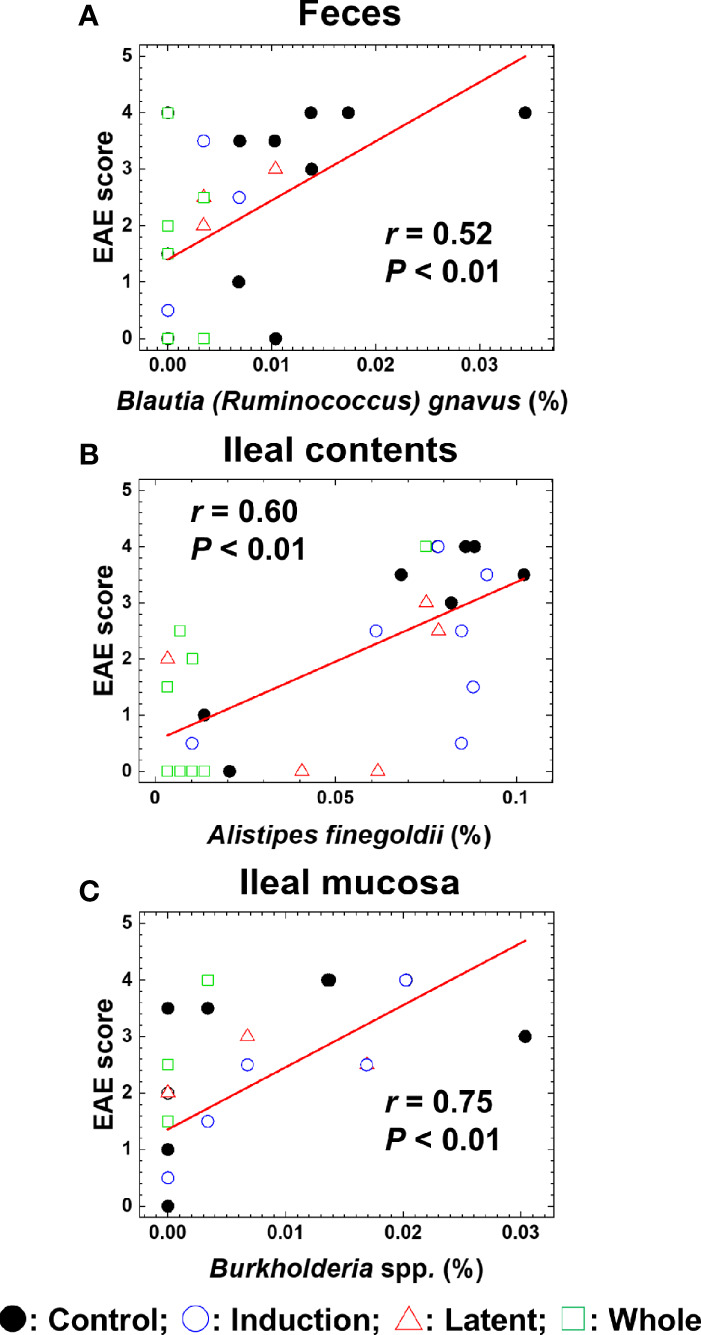
Pattern matching between the EAE scores and relative abundance of bacterial species. **(A–C)** We conducted pattern matching to correlate the EAE scores with the relative abundance of individual bacterial species in feces **(A)**, ileal contents **(B)**, and the ileal mucosa **(C)**. The figures are the representative bacterial species correlated with the EAE scores at the three anatomical sites. These bacterial species significantly decreased in the CMG-treated groups (Induction, blue circle; Latent, red triangle; and Whole, green square), compared with the control group (Control, black circle) (**Table 3**): **(A)** *Blautia (Ruminococcus) gnavus* in feces (Induction, Latent, and Whole < Control), **(B)** *Alistipes finegoldii* in ileal contents (Whole < Control), and **(C)** *Burkholderia* spp. in the ileal mucosa (Whole < Control).

We also conducted pattern matching between the relative abundance of individual bacterial species and EAE scores (**Figure 6**). Based on the *r* values (Mukaka, 2012), the EAE scores moderately correlated with the relative abundance of several species (five species in feces, five species in ileal contents, and two species in the ileal mucosa) and highly correlated with three species in the ileal mucosa (**Table 4**). Among these species, the relative abundance of *Ruminococcus bromii* and *Blautia (Ruminococcus) gnavus* in feces, *Turicibacter* sp. and *Alistipes finegoldii* in ileal contents, and *Burkholderia* spp. and *Azoarcus* spp. in the ileal mucosa was significantly different between the Control and CMG-treated groups (**Figure 6**, **Table 4**).

**Table 4:** Bacterial species correlated with the EAE scores*.

Correlation coefficient (*r*)	Feces		Ileal contents		Ileal mucosa	
	Bacterial species	*r*	Bacterial species	*r*	Bacterial species	*r*
***r* ≥ 0.5**	*Erysipelatoclostridium clostridium innocuum*	0.56	*Turicibacter* sp.**	0.66	*Burkholderia* spp.**	0.75
	Ruminococcus bromii**	0.55	*Bifidobacterium choerinum*	0.62	*Burkholderia* sp.	0.72
	*Porphyromonas* sp.	0.54	Alistipes finegoldii**	0.60	*Burkholderia ubonensis*	0.71
	Blautia (Ruminococcus) gnavus**	0.52	*Allobaculum stercoricanis*	0.56	*Azoarcus* spp.**	0.54
***r* ≥ –0.5**	*Atopostipes* sp.	–0.50	*Jeotgalicoccus halotolerans*	–0.66	*Barnesiella* spp.	–0.50

*Using pattern matching, we determined correlations between EAE scores and relative abundance of individual bacterial species. P < 0.01 (except Barnesiella spp., whose P value was < 0.05). Spearman’s rank correlation coefficient (r) = 0.50 to 0.70 (–0.50 to –0.70); moderate positive (negative) correlation and r = 0.70 to 0.90 (–0.70 to –0.90); high positive (negative) correlation (Mukaka, 2012).

**Bacterial species whose relative abundance in the CMG-treated groups was significantly different compared with the Control group by ANOVA, P < 0.05.

### 3.6 PICRUSt Predicts the CMG Effects on Bacterial Pathways

Lastly, we performed predictive metagenome analyses for microbiome data using the PICRUSt program, which predicts the pathways derived from the read counts of all bacteria in the microbiota involved in the pathways of interest. By comparing the numbers of pathways changed in the three CMG-treated groups, we did not find changes that were common in all CMG-treated groups or the three anatomical sites. However, we found that changes in the several pathways were common between the two CMG-treated groups and between the two anatomical sites (**Table 5**, **Supplemental Table 4**). CMG administration seemed to affect the bacterial pathways most in ileal contents and least in the ileal mucosa, the latter of which had no upregulated pathways by CMG administration. In fecal samples, five pathways were upregulated commonly in the Induction and Whole groups. In ileal contents, CMG administration upregulated ten pathways commonly in the Latent and Whole groups. By comparing the pathways changed by CMG administration among the three anatomical sites, we found that 14 pathways were commonly upregulated in feces and ileal contents in the Whole group, but not in the Induction or Latent group. In **Table 6**, we listed the pathways that were up- or down-regulated more than 2-fold in the three CMG-treated groups. We found several pathways related to immune response and inflammation, such as “viral myocarditis” and “arachidonic acid metabolism”.

**Table 5:** Numbers of pathways changed in the CMG-treated groups by PICRUSt.

Groups		Induction	Latent	Whole
**Feces**	Increased	5^a, b^	0	36^a, b, d^
	Decreased	0	0	2
**Ileal contents**	Increased	0	59^a, c^	26^a, c, d^
	Decreased	2	0	4
**Ileal mucosa**	Increased	0	0	0
	Decreased	0	0	23

Significantly changed pathways compared with the Control group (P < 0.05).

a Pathway commonly increased in feces of the Induction and Whole groups, and ileal contents of the Latent and Whole groups (n = 1).

1. Cell division.

b Pathways commonly increased in feces of the Induction and Whole groups (n = 5).

1. Cell division; 2. Glycosphingolipid biosynthesis - ganglio series; 3. 1,1,1-Trichloro-2,2-bis(4-chlorophenyl) ethane (DDT) degradation; 4. Toluene degradation; and 5. Glycan biosynthesis and metabolism.

c Pathways commonly increased in ileal contents of the Latent and Whole groups (n = 10).

1. Cell division; 2. Peroxisome; 3. Primary immunodeficiency; 4. Infectious diseases, pertussis; 5. Oxidative phosphorylation; 6. Biotin metabolism; 7. Folate biosynthesis; 8. Ubiquinone and other terpenoid-quinone biosynthesis; 9. β-Alanine metabolism; and 10. Membrane and intracellular structural molecules.

d Pathways commonly increased in feces and ileal contents of the Whole group (n = 15).

1. Cell division; 2. Peroxisome; 3. Infectious diseases, pertussis; 4. Citrate cycle (TCA cycle); 5. Oxidative phosphorylation; 6. Lipopolysaccharide biosynthesis; 7. Lipopolysaccharide biosynthesis proteins; 8. Biotin metabolism; 9. Ubiquinone and other terpenoid-quinone biosynthesis; 10. β-Alanine metabolism; 11. 1,1,1-Trichloro-2,2-bis(4-chlorophenyl) ethane (DDT) degradation; 12. Toluene degradation; 13. Adipocytokine signaling pathway; 14. Membrane and intracellular structural molecules; and 15. Glycan biosynthesis and metabolism.

**Table 6:** Predicted pathways up-or down-regulated (> 2-fold) in CMG-treated group.

Pathways	Fold change	Site	Group
1,1,1-Trichloro-2,2-bis(4-chlorophenyl)ethane (DDT) degradation	3.6	Feces	Induction
	4.3	Feces	Whole
	2.4	Ileal content	Whole
Cardiac muscle contraction	3.3	Feces	Whole
Parkinson’s disease	3.3	Feces	Whole
Influenza A	3.3	Feces	Whole
p53 signaling pathway	3.3	Feces	Whole
Colorectal cancer	3.3	Feces	Whole
Small cell lung cancer	3.3	Feces	Whole
Toxoplasmosis	3.3	Feces	Whole
Viral myocarditis	3.3	Feces	Whole
G protein-coupled receptors	2.5	Feces	Whole
	0.5	Ileal content	Whole
Photosynthesis - antenna proteins	0.5	Feces	Whole
Calcium signaling pathway	0.5	Feces	Whole
Germination	0.5	Ileal content	Whole
Arachidonic acid metabolism	0.5	Ileal mucosa	Whole
Bacterial chemotaxis	0.4	Ileal mucosa	Whole
Flagellar assembly	0.4	Ileal mucosa	Whole

Pathways listed by a program of Phylogenetic Investigation of Communities by Reconstruction of Unobserved States (PICRUSt) were based on KEGG database (https://www.genome.jp/kegg/).

We also determined the read counts of bacteria encoding β-glucuronidase between the CMG-treated and Control groups, since β-glucuronidase can be involved in CMG metabolism by converting CMG to free-form curcumin (Ozawa et al., 2017). We found no significant differences in the read counts of bacteria encoding β-glucuronidase between the CMG-treated and Control groups (**Supplemental Figure 12**).

## 4 Discussion

Although fecal samples are widely used for microbiome studies as a representative of the gut microbiota throughout the GI tract (Durbán et al., 2011), the microbial compositions have been shown to be different depending on the anatomical sites (Durbán et al., 2011; Durbán et al., 2012; Dieterich et al., 2018; Lee et al., 2018). Bacterial diversities have been reported to increase gradually along the GI tract (Lee et al., 2018) and differ between the GI lumen versus mucosa (Durbán et al., 2011; Durbán et al., 2012). Consistent with these findings, in the current study, we found that bacterial compositions in EAE mice differed not only among the three anatomical sites whose diversities were higher in feces than in ileum contents, but also between ileal contents and the ileal mucosa. This is the first study that compared the microbiota from the three anatomical sites in EAE.

To determine whether CMG administration could affect the microbiota at the three anatomical sites, correlating with the EAE severity, we conducted comparative analyses using the fecal, ileal content, and ileal mucosal samples. Although the reduced bacterial alpha diversity has been associated with the disease severities in many disease conditions (Bosshard et al., 2002; Deng et al., 2019), CMG administration modulated EAE (**Figure 1**) without increased alpha diversities of the microbiota (**Figure 2**). These results were compatible with the previous findings that the reduced bacterial alpha diversity was not observed in MS and its animal models (Cosorich et al., 2017; Omura et al., 2020).

Using PCA, we investigated whether CMG administration could alter the overall microbiome patterns among the three anatomical sites. We found that PC1 values of feces and ileal contents, but not the ileal mucosa, were significantly different between the CMG-treated versus Control groups (**Figure 3** and **Supplemental Figures 8**, **9**), suggesting that CMG administration altered the microbiota compositions in feces and ileal contents. This could be due to the distinct metabolism of free-form curcumin at the three anatomical sites. In general, following oral administration of free-form curcumin, its conjugation takes place mainly in the liver and some in the intestine; through the enterohepatic circulation, conjugated curcumin reaches the intestine with bile, where deconjugation occurs by β-glucuronidase (Ozawa et al., 2017). Since the β-glucuronidase activity contributes to converting the inactive conjugated curcumin to the active free-form of curcumin, the distribution of free-form curcumin in tissues has been highest in the intestine followed by the spleen and the liver after intraperitoneal administration of free-form curcumin in mice (Pan et al., 1999). On the other hand, in the colon cancer-bearing mouse xenograft models, mice with intraperitoneal CMG administration exhibited the high levels of free-form curcumin in the tumor tissue, comparing with serum and major organs (heart, liver, and spleen) (Ozawa-Umeta et al., 2020). In this study, free-form curcumin either in the GI tract or in the blood derived from CMG could directly affect the gut microbiota. Since CMG altered microbiome of the contents of the GI tract, i.e., feces and ileal contents, but not the ileal mucosa, free-form curcumin in the GI tract, which was likely generated by deconjugation of CMG in the GI luminal site, seemed to directly affect the microbiota present in the lumen; curcumin in the GI lumen may have no contact with mucosal bacteria.

PICRUSt analyses demonstrated that the levels of bacteria encoding β-glucuronidase were higher in fecal samples than in the ileal content/mucosa samples; this is also consistent with the findings that fecal microbiota was affected most by administration with CMG, which can be deconjugated by β-glucuronidase released in the GI lumen. Although we found no differences in bacteria encoding β-glucuronidase between ileal contents and the ileal mucosa in PICRUSt analyses, in the GI tract, free enzymes might be released higher in the lumen than in the mucosa. Thus, the conversion of CMG to free-form curcumin in the GI tract may directly affect the microbiome; the concentration of free-form curcumin may be lowest in the ileal mucosa among the three anatomical sites. Alternatively, free-form curcumin in the blood may also have no contact with mucosal bacteria since the mucus layer is present between the intestinal epithelial surface and mucosal bacteria. In theory, although free-form curcumin in the blood can affect the immune system, influencing the composition of the gut microbiota, this influence seemed to be limited since only a small number of bacterial species changed their relative abundance in the ileal mucosa (**Table 3**) without changes in overall microbiota (**Figure 3** and **Supplemental Figures 8**, **9**).

Associations between the gut microbiomes and disease activities have been reported to differ among the distinct anatomical sites as well as the content versus mucosal samples. For example, in the 2,4,6-trinitrobenzenesulfonic acid (TNBS)-induced colitis model in mice, the alterations in the colonic mucus microbiomes more closely correlated with the disease severity than those in the fecal and cecal microbiomes (Kozik et al., 2019). In the current study, using PCA, we demonstrated that CMG administration altered overall bacterial compositions in feces and ileal contents, but not in the ileal mucosa. Then, using pattern matching, we found that PC1 values of microbiome data in ileal contents and mucosa, but not in feces, correlated with the clinical and neuropathological scores in EAE. Thus, among the three anatomical sites, the altered overall microbiota in ileal contents by CMG administration could contribute to the modulation of EAE. On the other hand, dysbiosis in the ileal mucosa seemed to be associated with the EAE severity regardless of CMG administration. Our results were consistent with previously published observations that the ileal contents/mucosa microbiota direct the differentiation of Th17 cells in mice (Ivanov et al., 2008; Farkas et al., 2015). In human MS, microbiota alterations in the small intestinal mucosa have been reported to correlate with disease activity (Cosorich et al., 2017).

Previously, we demonstrated that, in a viral model for MS, Theiler’s murine encephalomyelitis virus (TMEV) infection, alterations in individual bacterial genera, but not changes in the overall gut microbiome, were associated with immune-gene expressions in the CNS (Omura et al., 2020). Similarly, in the current study, although CMG administration affected the overall microbiota patterns in feces and ileal contents, but not the ileal mucosa, pattern matching demonstrated that six bacterial species (two species at each anatomical site, **Table 4**) correlated moderately or highly with the EAE scores among the significantly decreased bacterial species by CMG administration: *Ruminococcus bromii* and *Blautia (Ruminococcus) gnavus* in feces, *Turicibacter* sp. and *Alistipes finegoldii* in ileal contents, and *Burkholderia* spp. and *Azoarcus* spp. in the ileal mucosa. Thus, the decreases in individual bacterial species by CMG administration might contribute to suppression of EAE (Shen et al., 2017; Zhang et al., 2017; Peterson et al., 2018; Gandy et al., 2019; Kozik et al., 2019).

Among the six species, *Ruminococcus bromii* belongs to the phylum *Firmicutes*, family *Ruminococcaceae*, and produces short-chain fatty acids (SCFAs) (Park et al., 2019; Yao et al., 2020). SCFAs have been reported to contribute to the pathogenesis of EAE by inducing pro-inflammatory responses, including Th17 cells, depending on the SCFA receptors (Park et al., 2019). On the other hand, SCFAs have also been reported to be protective in EAE by enhancing anti-inflammatory responses, including Tregs (Park et al., 2019; Yao et al., 2020). *Blautia (Ruminococcus) gnavus* belongs to the phylum *Firmicutes*, family *Lachnospiraceae*, and genus *Blautia* (Hansen et al., 2013). The genus *Blautia* has been reported to increase in feces of patients with relapsing-remitting MS (Chen et al., 2016). Furthermore, increased abundance of *Blautia (Ruminococcus) gnavus* positively correlated with disease activity in patients with IBD and systemic lupus erythematosus (Breban et al., 2017; Azzouz et al., 2019).

*Turicibacter* sp. belongs to the phylum *Firmicutes*, family *Erysipelotrichaceae*, and has been reported as a pro-inflammatory taxon (Bosshard et al., 2002; Ma et al., 2018). For example, Miyauchi et al. demonstrated that a newly isolated bacterium OTU0002 *Erysipelotrichaceae* exacerbated EAE with enhanced MOG_35-55_-specific Th17 responses by increasing serum amyloid A and IL-23 production (Miyauchi et al., 2020). *Turicibacter sangunis* was isolated from patients with acute appendicitis (Bosshard et al., 2002). *Alistipes finegoldii* belongs to the phylum *Bacteroidetes*, family *Rikenellaceae*, and has been reported to be a potential SCFA producer (Dziarski et al., 2016; Parker et al., 2020). *Alistipes* could play a pro-inflammatory role in cardiovascular disease, although the role of *Alistipes* in IBD is controversial (Parker et al., 2020). *Burkholderia* spp. belongs to the phylum *Proteobacteria*, family *Burkholderiaceae*, and some of the species caused a severe pulmonary disease (Urban et al., 2004; Wiersinga et al., 2008). Experimentally, *Burkholderia* induced inflammation in the lungs due to the production of proinflammatory cytokines in mice (Wiersinga et al., 2008). *Azoarcus* sp. belongs to the phylum *Proteobacteria*, family *Zoogloeaceae*, and has only been reported in antibiotics-induced diarrhea in experimental mice which was associated with dysbiosis in the intestine. Here, antibiotics treatment altered the gut microbiota including the abundance of *Azoarcus* (Long et al., 2017).

In this study, we used PICRUSt analyses to predict whether CMG administration affected multiple bacterial pathways depending on the CMG administration schedule and the anatomical sites. Although we found that changes in the several pathways were common between the CMG-treated groups and between the anatomical sites, we could not find changes that were common in all CMG-treated groups or the three anatomical sites (**Table 5** and **Supplemental Table 4**). Since biological changes in the gut bacteria by polyphenol have been reported to affect the immune system potentially (Kawabata et al., 2018), we examined the biological pathway changes in the CMG-treated groups. Although we did not find bacterial pathways that have been demonstrated to affect the host’s systemic immune responses, including the production of SCFAs (Takewaki and Yamamura, 2021), we found several pathways potentially related to immune responses, including “viral myocarditis” and “arachidonic acid metabolism” in the list of upregulated pathways more than 2-fold in the three CMG-treated groups (**Table 6**). We also found that two bacterial pathways related to immune responses (“primary immunodeficiency” and “infectious diseases, pertussis”) were commonly increased in the Latent and Whole groups whose EAE scores were less severe than controls. Although PICRUSt predicted neither substantial pathways altered commonly in all CMG-treated groups, nor pathways directly associated with the EAE pathophysiology, more accurate metagenome sequencing analyses may lead to a discovery of metabolome changes by CMG administration.

In EAE using IL-10 transgenic mice and recombinant IL-10 administration, the beneficial effects of IL-10 have been reported, such as inhibition of encephalitogenic T cell responses (Bettelli et al., 1998; Cua et al., 1999); lack of IL-10 exacerbated EAE with larger amounts of pro-inflammatory cytokines, including IFN-γ (Bettelli et al., 1998). IL-10 has been shown to have anti-inflammatory effects in EAE; there have been several reports that curcumin regulated EAE by increasing the levels of IL-10. For example, Mohajeri et al. demonstrated both prophylactic and therapeutic effects of curcumin in an EAE model using Lewis rats with increased IL-10 gene expression (Mohajeri et al., 2015). Kanakasabai et al. reported that curcumin-treated C57BL/6 mice exhibited less severe MOG_35-55_-induced EAE scores and higher levels of IL-10 expression compared with the control mice (Kanakasabai et al., 2012). Similarly, IL-4 has been demonstrated to play a regulatory role in EAE (Fernando et al., 2014). In this study, we demonstrated the beneficial effects of curcumin in MOG_35-55_-EAE, where CMG-treated mice, particularly those in the Latent and Whole groups, exhibited lower clinical and neuropathological scores; the Latent and Whole groups had slightly higher levels of anti-inflammatory IL-4 and IL-10 productions (**Supplemental Figure 2**). Thus, the enhanced IL-4 and IL-10 productions by CMG administration may contribute to the modulation of EAE, only to some extent. The poor association between anti-inflammatory IL-4 and IL-10 levels and the severities of EAE by CMG administration could be due to the enhancement of increased levels of pro-inflammatory IFN-γ and IL-17 in these two CMG-treated groups. Here, it should be noted that IFN-γ has been shown to be beneficial in some EAE models by regulating IL-17 production (Martinez et al., 2014); the enhanced IFN-γ production in the Latent and Whole groups might play a protective role in this EAE model.

Immunologically, curcumin has also been known to regulate the proliferation of lymphocytes. When Kanakasabai et al. cultured splenic cells from MOG_35-55_-EAE mice *in vitro* in the presence of curcumin, the lymphoproliferative responses to the MOG_35-55_ peptide were decreased in a dose-dependent manner (Kanakasabai et al., 2012). On the other hand, splenic cells from EAE mice injected with intraperitoneal curcumin had similar levels of MOG_35-55_-specific lymphoproliferation to those from the control EAE mice. Consistent with the Kanakasabai’s findings, when we tested whether curcumin could directly inhibit the MOG_35-55_-specific lymphoproliferation using lymphocytes from the spleen or lymph nodes from 2D2 T cell receptor (TCR)^MOG^ transgenic mice in the presence of curcumin, lymphoproliferation was suppressed by curcumin in a dose-dependent manner (**Supplemental Figures 13C, D**) (Tsunoda et al., 2009). Similarly, curcumin directly inhibited the proliferation of a T cell line more efficiently than that of neuronal cells *in vitro* (**Supplemental Figures 13A, B**). On the other hand, we found no differences in the MOG_35-55_-specific lymphoproliferation among the CMG-treated and Control groups when we used lymphocytes isolated from EAE mice (**Supplemental Figure 3**). These findings suggest that curcumin potentially has direct suppressive effects on lymphocytes; curcumin could directly affect the MOG_35-55_-specific T cell proliferation *in vivo*, resulting in the modulation of the MOG_35-55_-EAE model. This may also explain that the efficacy of CMG administration differed depending on the administration schedule; mice receiving CMG throughout the course (Whole group) had the lowest cumulative scores of MOG_35-55_-induced EAE among the CMG-treated groups. Suppression of CNS inflammation by curcumin has been reported in EAE (Natarajan and Bright, 2002; Xie et al., 2009; Mohajeri et al., 2015); we also demonstrated decreased inflammation and demyelination in the spinal cord of chronic monophasic EAE by CMG administration.

The limitation of the current study is the modest effects of CMG in MOG-induced EAE using C57BL/6 mice. Thus, we investigated whether CMG could modulate another EAE model, by sensitizing a different mouse strain, SJL/J mice, with the PLP_139-151_ peptide. Since PLP-sensitized mice develop relapsing-remitting EAE, this model allowed us to test whether CMG could exert the similar beneficial effects on the different disease courses in the different mouse strains. Unexpectedly, however, we did not find the amelioration of CMG-treated mice with PLP-induced EAE clinically or neuropathologically (**Supplemental Table 5** and **Supplemental Figures 14A, B**). To clarify the discrepancy between MOG-induced EAE and PLP-induced EAE, we examined whether a lack of the treatment effect could be due to a lack of suppression of PLP-specific T cell proliferation by curcumin using lymphocytes isolated from PLP-induced EAE mice. Intriguingly, we found that the PLP-specific lymphoproliferation was suppressed by curcumin in a dose-dependent manner (**Supplemental Figures 14C, D**). Here, although the precise mechanism for the different effects of CMG on the two EAE models is unclear, this was consistent with the previous reports that the efficacy of curcumin was different among the EAE types and animal species (Verbeek et al., 2005; Mohajeri et al., 2015). The different efficacy of CMG in these animal models could be due to several factors including: variation in the microbiota among different mouse strains, i.e., C57BL/6 mice versus SJL/J mice (Verbeek et al., 2005; Mohajeri et al., 2015), whose major bacterial phyla of the gut microbiota are *Verrucomicrobia* and *Tenericutes* phyla, respectively (Gandy et al., 2019). This may also be due to the distinct disease courses and pathomechanisms between the two EAE models. MOG-induced EAE mice develop a monophasic disease course; PLP-induced EAE mice exhibit a relapsing-remitting disease course. Myelin-specific CD4^+^ T cells, but not CD8^+^ T cells or antibodies, have been shown to mediate both MOG_35-55_-induced and PLP_139-151_-induced EAE (Tsunoda et al., 1998; Fernando et al., 2014; Martinez et al., 2014). In the MOG-induced EAE model, autoreactive T cell responses to the sensitized antigen MOG contribute to the pathomechanism. On the other hand, although the first attack in the PLP-induced EAE model is also caused by encephalitogenic T cells against the sensitized antigen PLP, the generation of encephalitogenic T cells that react to different myelin antigens has been proposed as the pathomechanism of relapses (known as “epitope spreading”) (McRae et al., 1995).

In the future, CMG treatment is worth testing in MS models, using two different administration schedules. First, we treated mice in the Induction group on days 0–4 to see whether CMG could affect the priming of MOG-specific T cells. Since there were no differences in MOG-specific lymphoproliferation, CMG treatment seemed not to alter the priming by directly affecting lymphocytes and antigen-presenting cells. Although this induction treatment schedule has often been regarded as a prophylactic treatment in most EAE studies, earlier CMG administration, a few weeks before EAE induction, will give more insight into the beneficial effect of CMG as an alternative prophylactic treatment. Here, if CMG is administered a few weeks before EAE induction, it can change the compositions of gut microbiota, altering the molecules/cells of both humoral and cellular immune effectors, including IgA antibody, Th17 cells, and Tregs. Thus, the early CMG prophylactic treatment might affect MOG-specific immune responses, suppressing EAE more efficiently. Second, we discontinued CMG treatment during the late chronic stage of PLP-induced EAE (**Supplemental Figure 14**); since the second and third relapsing/remissions of PLP-EAE occur randomly in each mouse and do not synchronize among mice, PLP-EAE is often inappropriate to assess the efficacy of drugs. However, CMG can be administered at the late chronic stage of other MS models, such as the TMEV model, in which similar disease progression occurs among most experimental mice.

In conclusion, we found that CMG administration was safe and modulated an autoimmune model of MS by altering the microbiomes in the feces and ileal contents, but not in the ileal mucosa. The microbiome of ileal contents as well as several individual bacterial species in the three anatomical sites correlated with the EAE severities, suggesting that CMG might suppress EAE through alteration of the microbiota. This report would provide insight into how CMG potentially affects the gut microbiota depending on the anatomical sites, which associates with the EAE pathophysiology.

## Data Availability Statement

The original contributions presented in the study are publicly available in NCBI using accession number PRJNA688384.

## Ethics Statement

The animal study was reviewed and approved by The Institutional Animal Care and Use Committee of Kindai University Faculty of Medicine.

## Author Contributions

IT, HK, and KN conceived and supervised the project. IT and HK designed the experiments. SK, SO, and FS conducted the experiments and data analyses. SK, SO, FS, HK, and IT wrote the manuscript. All authors contributed to the article and approved the submitted version.

## Funding

This work was supported by the Grant-in-Aid for Scientific Research on Innovative Areas “Frontier Research on Chemical Communications” [17H06400 (HK and IT), 17H06401 (HK), and 17H06404 (IT)], Grant-in-Aid for Scientific Research (C) KAKENHI from the Japan Society for the Promotion of Science [JP19K08569 (SO), JP20K07433 (FS), and JP20K07455 (IT)], and Novartis Pharma Research Grants (SO and IT).

## Conflict of Interest

HK owns equity and is a scientific consultant for Therabiopharma.

The remaining authors declare that the research was conducted in the absence of any commercial or financial relationships that could be construed as a potential conflict of interest.

This study received funding from Novartis Pharma. The funder was not involved in the study design, collection, analysis, interpretation of data, the writing of this article or the decision to submit it for publication.

## Publisher’s Note

All claims expressed in this article are solely those of the authors and do not necessarily represent those of their affiliated organizations, or those of the publisher, the editors and the reviewers. Any product that may be evaluated in this article, or claim that may be made by its manufacturer, is not guaranteed or endorsed by the publisher.
