# Corrigendum: Curcumin β-D-Glucuronide Modulates an Autoimmune Model of Multiple Sclerosis with Altered Gut Microbiota in the Ileum and Feces

**DOI:** 10.3389/fcimb.2022.855411

**Published:** 2022-03-01

**Authors:** Sundar Khadka, Seiichi Omura, Fumitaka Sato, Kazuto Nishio, Hideaki Kakeya, Ikuo Tsunoda

**Affiliations:** ^1^ Department of Microbiology, Kindai University Faculty of Medicine, Osaka, Japan; ^2^ Department of Genome Biology, Kindai University Faculty of Medicine, Osaka, Japan; ^3^ Graduate School of Pharmaceutical Sciences, Kyoto University, Kyoto, Japan

**Keywords:** bioinformatics, animal model, pattern matching, PICRUSt analysis, bacterial taxonomy, Alpha diversity, confidence interval, histology

**Error in Figure/Table**

In the published article, there was a mistake in the PDF article as published. **Figure 5** with its legend is missing from the PDF article, although the HTML article has included **Figure 5**. The missing **Figure 5** with its legend appears below.

**Figure 5:**
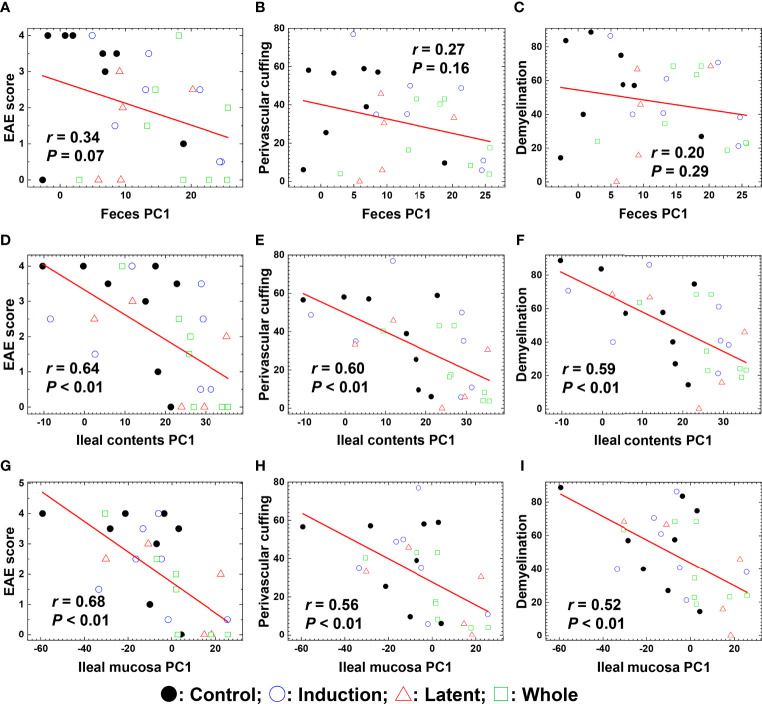
Pattern matching of the microbial PC1 values with the clinical and pathological scores of EAE at the three anatomical sites: feces, ileal contents, and the ileal mucosa. **(A–I)** We harvested samples from the three CMG-treated (Induction, blue circle; Latent, red triangle; and Whole, green square) and control groups (Control, black circle). The microbial PC1 values of ileal contents and the ileal mucosa, but not feces, significantly correlated with the EAE scores **(A, D, G)**, perivascular cuffing (i.e., inflammation) scores **(B, E, H)**, and demyelination scores **(C, F, I)** (*P* < 0.01).

The authors apologize for this error and state that this does not change the scientific conclusions of the article in any way.

## Publisher’s Note

All claims expressed in this article are solely those of the authors and do not necessarily represent those of their affiliated organizations, or those of the publisher, the editors and the reviewers. Any product that may be evaluated in this article, or claim that may be made by its manufacturer, is not guaranteed or endorsed by the publisher.

