# Supplementary material for: Curcumin β-D-Glucuronide Modulates an Autoimmune Model of Multiple Sclerosis with Altered Gut Microbiota in the Ileum and Feces
Source: Front Cell Infect Microbiol. 2021 Dec 3;11:772962. doi: 10.3389/fcimb.2021.772962 (PMC8677657; doi:10.3389/fcimb.2021.772962)
Supplement: Supplementary file 1 [file DataSheet_1.zip › V53. Supplementary file.docx]

**Supplemental Data**

**Table of contents**

**Supplemental Figure 1.** Histological examination of curcumin monoglucuronide (CMG) in myelin oligodendrocyte glycoprotein (MOG)_35-55_-induced experimental autoimmune encephalomyelitis (EAE). ...2

**Supplemental Figure 2.** Cytokine profiles of the Control and CMG-treated groups with MOG-EAE ...3

**Supplemental Figure 3.** Lymphoproliferative responses to MOG of the Control and CMG-treated groups with MOG-EAE ...4

**Supplemental Figure 4.** Analyses of bacterial alpha diversities of microbiome from three anatomical sites (feces, ileal contents, and the ileal mucosa) ...5

**Supplemental Figure 5.** Principal component analysis (PCA) of microbiome data at three taxonomical bacterial levels (phylum, genus, and species) from three anatomical sites ...6

**Supplemental Figure 6.** Principal component (PC)1 values in PCA of microbiome data at the three taxonomical bacterial levels (phylum, genus, and species) from three anatomical sites ...7

**Supplemental Figure 7.** PC2 values in PCA of microbiome data at the three taxonomical bacterial levels (phylum, genus, and species) from three anatomical sites ...8

**Supplemental Figure 8.** PCA of microbiome data at the phylum level from the Control and CMG-treated groups ...9

**Supplemental Figure 9.** PCA of microbiome data at the genus level from the Control and CMG-treated groups ...10

**Supplemental Figure 10.** Differences of fecal microbiota between control and EAE groups ...11

**Supplemental Figure 11.** Relative abundance of bacteria from three anatomical sites ...12

**Supplemental Figure 12.** Predictive read count levels of β-glucuronidase using microbiome data of Control and CMG-treated groups from three anatomical sites by PICRUSt ...13

**Supplemental Figure 13**. Effects of curcumin *in vitro* on neuronal and lymphoid cell lines ...14

**Supplemental Figure 14.** Effects of CMG on myelin proteolipid protein(PLP)_139-151_-induced EAE ...15

**Supplemental Table 1.** Effects of CMG on clinical courses and immune cells of mice with MOG_35-55_-induced EAE ...16

**Supplemental Table 2.** Compositional differences of microbiota between three anatomical sites ...17

**Supplemental Table 3.** Relative abundance data on the phylum, genus, and species levels ...Supplemental Excel File 1

**Supplemental Table 4.** Pathways commonly changed between the Control and CMG-treated groups from three anatomical sites ...Supplemental Excel File 1

**Supplemental Table 5.** Effects of CMG on the incidence of EAE in the mice sensitized with PLP_139-151_-induced EAE ...18

**
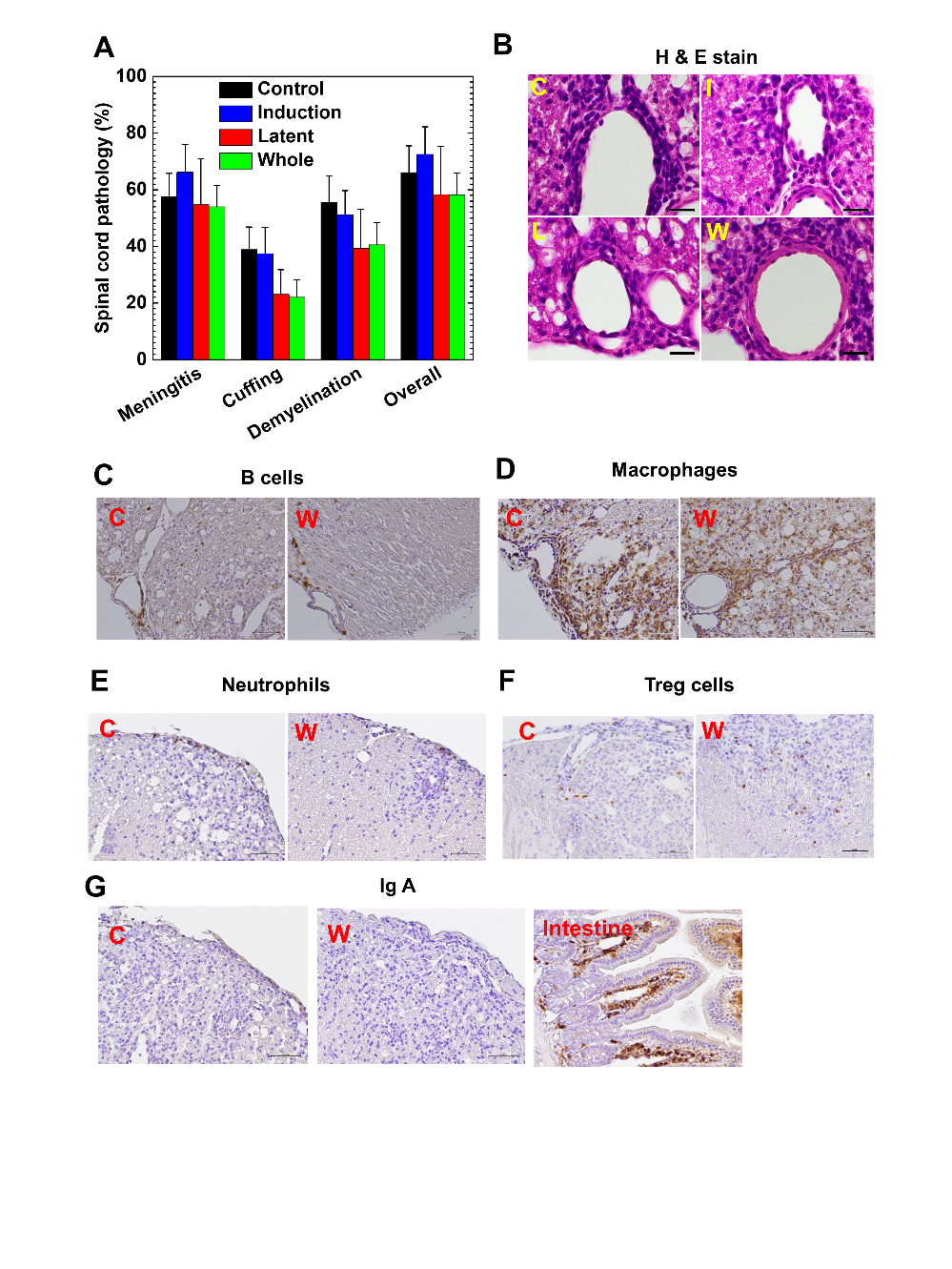
**

**Supplemental Figure 1.** Histological examination of curcumin monoglucuronide (CMG) in myelin oligodendrocyte glycoprotein (MOG)_35-55_-induced experimental autoimmune encephalomyelitis (EAE). We induced EAE in C57BL/6 mice by sensitizing with the MOG_35-55_ peptide. Mice were divided into four groups: the Control (C), Induction (I), Latent (L), and Whole (W) groups, where the groups of mice were treated with CMG on days 0-4 (Induction), on days 11-15 (Latent), or throughout the course (Whole). The control mice (Control) were treated with phosphate-buffered saline (PBS). Values are the mean ± standard error (SE). (**A**) We stained the spinal cord sections from EAE mice with Luxol fast blue to visualize the myelin. We compared demyelination, meningitis, perivascular inflammation (cuffing), and overall pathology scores between the CMG-treated and Control groups. Pathological changes in the spinal cords reflected the clinical scores when the CNS tissues were harvested (i.e. day 35); the Latent and Whole groups had lower demyelination, cuffing, and overall pathology scores in the spinal cords than the Control group. Neuropathology scores in the spinal cords were similar between the Induction and Control groups. Neuropathology scoring of the spinal cords was conducted in a blinded fashion. For scoring of the spinal cords section, we divided each transverse section into four quadrants consisting of the ventral, dorsal, and each lateral funiculus. Any quadrant containing meningitis, perivascular cuffing, inflammation, or demyelination was given a score of 1 in that pathological class. The total number of positive quadrants from all sections for each pathologic class was determined, then divided by the total number of quadrants present on the slide and multiplied by 100 to give the percentage involvement for each pathologic class. We also determined the overall pathology score by counting the number of quadrants containing any lesions (meningitis, perivascular cuffing, or demyelination) in the sections. Results indicate the mean + SE. (**B**) Hematoxylin and eosin stain (scale bar = 20 µm). There were no differences in the numbers of mononuclear cells (MNCs) versus polymorphonuclear cells (PMNs) in CNS cellular infiltrates, which were composed of MNCs in all groups. (**C-G**) Immunohistochemistry for B220^+^ B cells (**C**), F4/80^+^ macrophages (**D**), Ly-6G^+^ neutrophils (**E**), Foxp3^+^ regulatory T (Treg) cells (**F**). We found similar infiltrations of B cells, macrophages, neutrophils, and Treg cells among the control and CMG-treated groups. The stained sections are representatives of seven to nine mice per group. (**G**) IgA^+^ cells were not detected in the spinal cord from any groups, although IgA^+^ cells were observed in the intestine. Scale bar = 50 µm. (C = Control, I = Induction, L = Latent, and W = Whole groups)

**
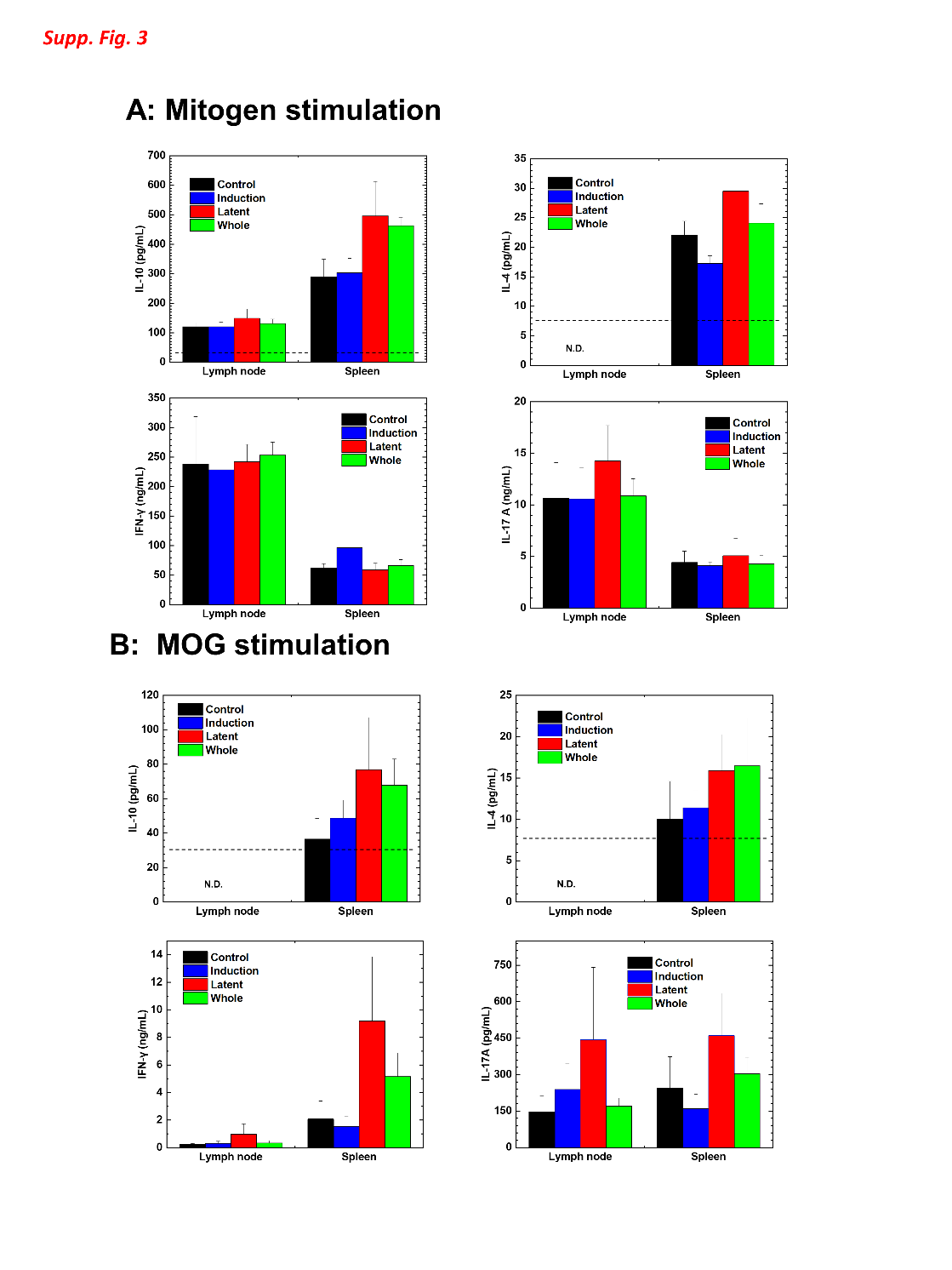
**

**Supplemental Figure 2.** Cytokine profiles of the CMG-treated and control EAE groups. We quantified the levels of interleukin (IL)-10; IL-4; interferon (IFN)-γ; and IL-17 by enzyme-linked immunosorbent assays (ELISAs). (**A**) In mitogen stimulation, although the Latent group tended to produce higher amounts of IL-10 compared with the Control group (*P* < 0.1), there were no statistical differences in the levels of IL-4, IFN-γ, or IL-17 production among the four groups. In all groups, IL-10 and IL-4 levels were higher in spleen cultures than in lymph node cultures; IFN-γ and IL-17 levels were higher in lymph node cultures than in spleen cultures. (**B**) In MOG stimulation, all four cytokine levels were lower than mitogen stimulation. Although splenic IFN-γ production tended to be higher in the latent group (*P* < 0.1) compared with the Control group, there were no statistical differences in the levels of the other cytokines among the four groups. No cytokines were detectable in spleen or lymph node cultures without stimulation. Values are the mean + SE of four pools of splenic mononuclear cells (MNCs) and lymph node cells. The detection limits of IL-10 and IL-4 were shown in the dotted lines: IL-10, 31.3 pg/mL; IL-4, 7.8 pg/mL; IFN-γ, 31.3 pg/mL; and IL-17, 15.6 pg/mL. We killed mice 5 weeks post induction (p.i.), and harvested the spleens and inguinal lymph nodes, and mashed the tissues on a metal mesh with 50-μm pores. Splenic MNCs were isolated using Histopaque^®^-1083. We cultured splenic MNCs and lymph node cells at a concentration of 8 × 10^6^ cells/well in 6-well plates in RPMI-1640 medium supplemented with 10% fetal bovine serum (FBS), 2 mM L-glutamine, 50 mM β-mercaptoethanol, and a 1% antibiotics solution containing 10,000 U/mL penicillin and 10,000 μg/mL streptomycin. We stimulated the cells with 5 µg/mL of the mitogen concanavalin A (ConA) or 50 µg/mL of the MOG_35−55_ peptides for 2 days. The culture supernatants were harvested and stored at ‒80°C until examined. We quantified the amounts of IL-10, IL-4, IFN-γ, and IL-17 in the culture supernatants using the ELISA kits, according to the manufacturer’s instructions. We conducted ELISA in duplicate using 96-well plates. N.D., not detectable.


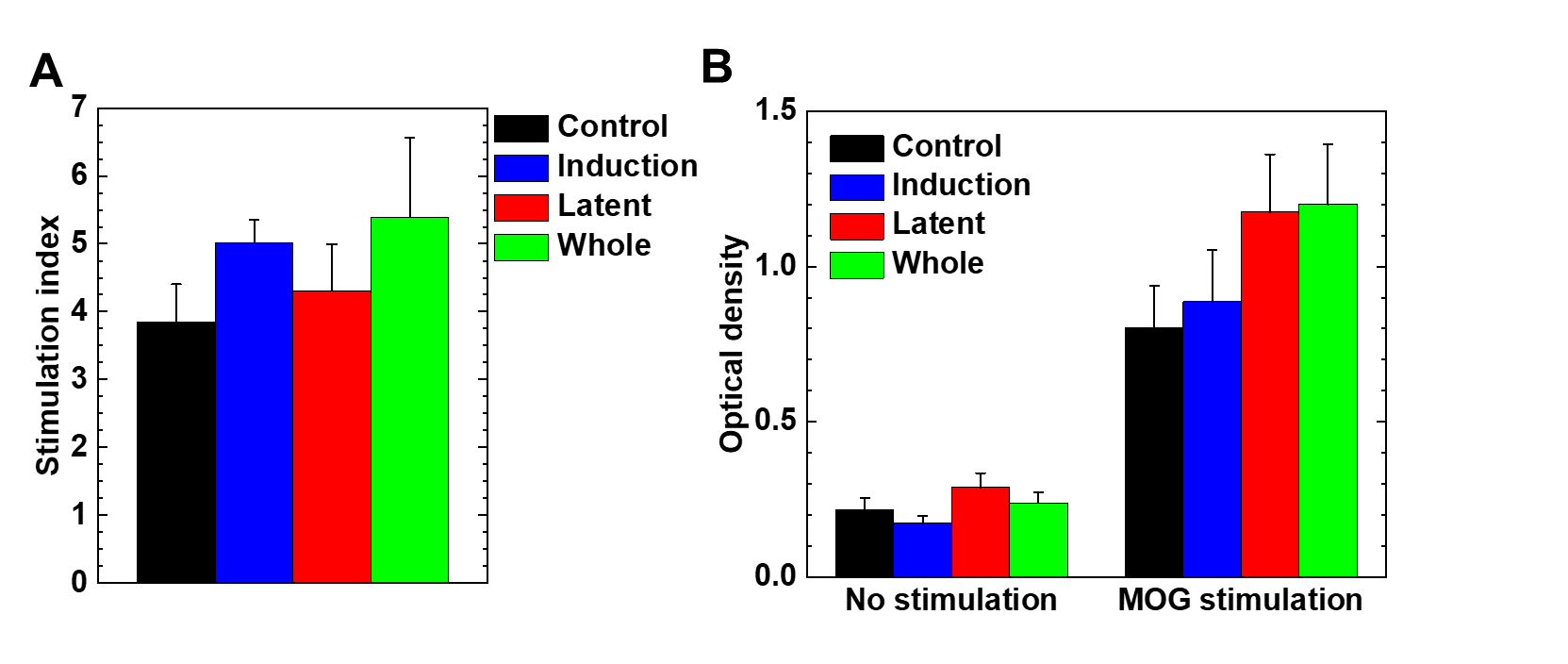


**Supplemental Figure 3**. Lymphoproliferative responses to MOG using MNCs isolated from the CMG-treated and control EAE groups. (**A**) All four groups had the substantial levels of MOG-specific proliferation (stimulation index >3) without significant differences among the groups. (**B**) Although MOG-specific lymphoproliferative responses were higher in the Latent and Whole groups than in the Control group, there were no statistical differences between the groups. MOG-specific lymphoproliferation was mediated by CD4^+^ T cells, but not CD8^+^ T cells, in our experimental system (Fernando et al., Int J Mol Sci, 2014). We isolated MNCs from spleens of EAE mice using Histopaque 1038. MNCs were cultured in RPMI-1640 medium, supplemented with 10% FBS, 2 mM L-glutamine, 50 mM β-mercaptoethanol, and 1% antibiotic-antimycotic solution at 2 × 10^5^ cells/well in 96-well plates. To assess the level of lymphoproliferative responses, MNCs were cultured in the presence or absence of 50 µg/mL MOG_35-55_ peptide for 5 days. For the last 24 hours, we added 20 µl/well of Cell Counting Kit-8 reagent (CCK-8, Dojindo Molecular Technologies, Inc., Rockville, US) to wells of 96-well plates and incubated the plates at 37°C. The CCK-8 detected the dehydrogenase activity in viable cells. The optical density (O.D.) was measured at 450 nm using a Wallac ARVO 1420 multilabel counter. All cultures were performed in triplicate and the data were expressed as the mean + SE of stimulation indexes (MOG stimulation/No stimulation) or O.D. of four sample pools of spleens from seven to nine mice per group. Each sample pool was composed of one to three spleens.


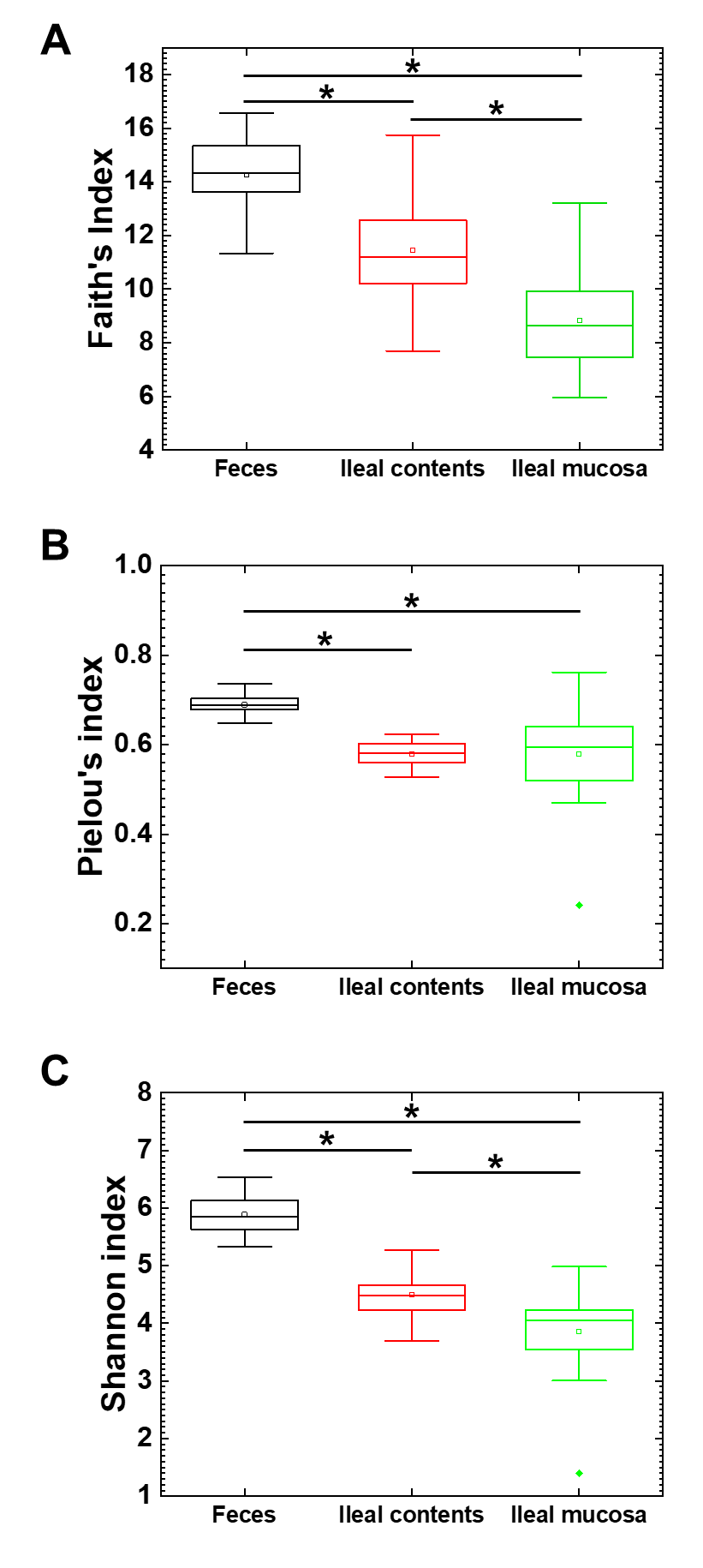


**Supplemental Figure 4.** Analyses of bacterial alpha diversities of microbiome from three anatomical sites. Using QIIME 2, we compared the number of genera, evenness, and combination of them by the Faith’s phylogenetic diversity (**A**), Pielou’s evenness (**B**), and Shannon (**C**) indexes, respectively, between three anatomical sites: feces (black), ileal contents (red) and the ileal mucosa (green). (**A**) The Faith’s phylogenic diversity index was significantly different among the three anatomical sites (**P* < 0.05, ANOVA). (**B**) The Pielou’s eveness index was significantly different between faces versus ileal contents, and feces versus the ileal mucosa (**P* < 0.05, ANOVA). (**C**) The Shannon index was significantly different among the three anatomical sites (**P* < 0.05, ANOVA). Total sample number was 28 per each anatomical site (Control, n = 8; Induction, n = 7; Latent, n = 5; and Whole, n = 8).

**
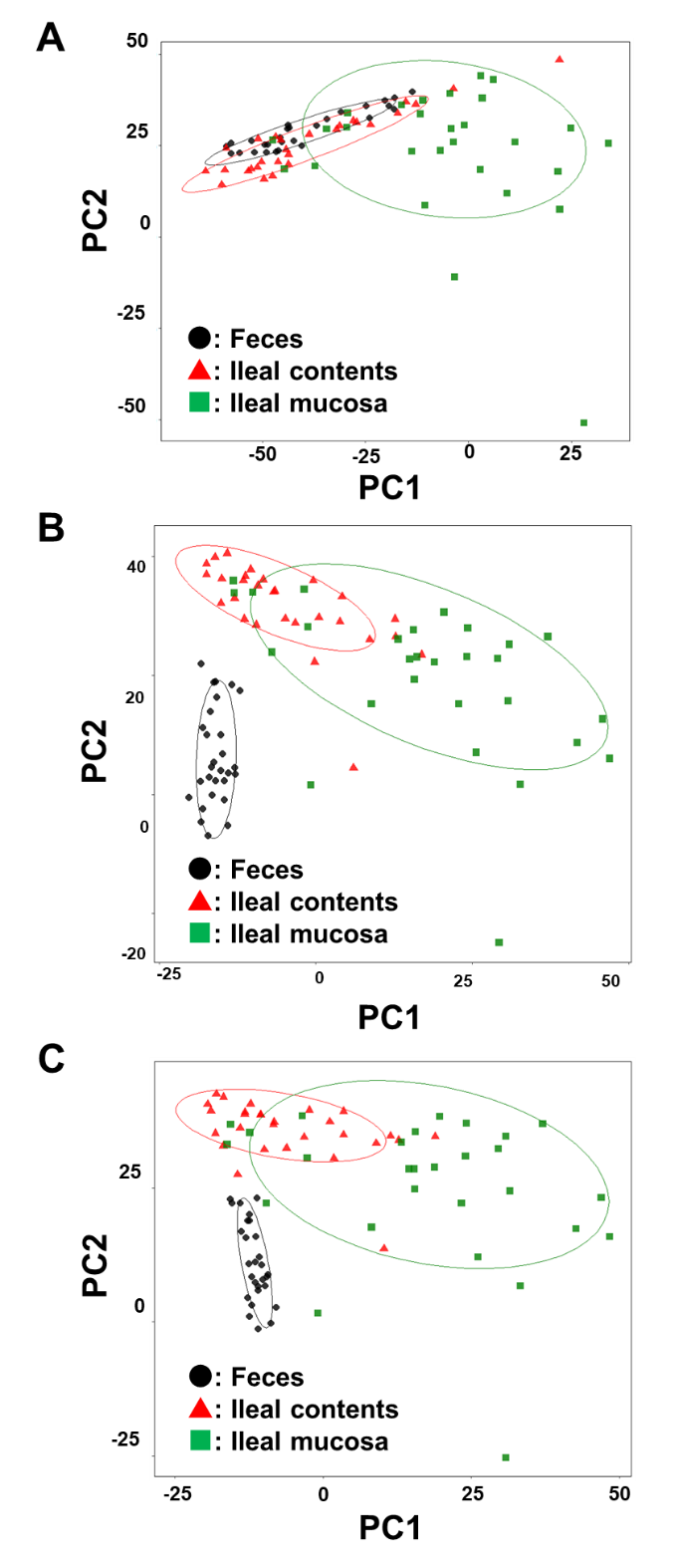
**

**Supplemental Figure 5.** Principal component analysis (PCA) of microbiome data from the three anatomical sites: feces (black), ileal contents (red), and the ileal mucosa (green). We conducted PCA using microbiome data at the phylum (**A**), genus (**B**) and species (**C**) levels. Ellipses indicated an 80% confidence interval of each group. Principal component (PC)1 values significantly different between feces versus the ileal mucosa, and ileal contents versus the ileal mucosa (**P* < 0.05) at the three taxonomical bacterial levels.


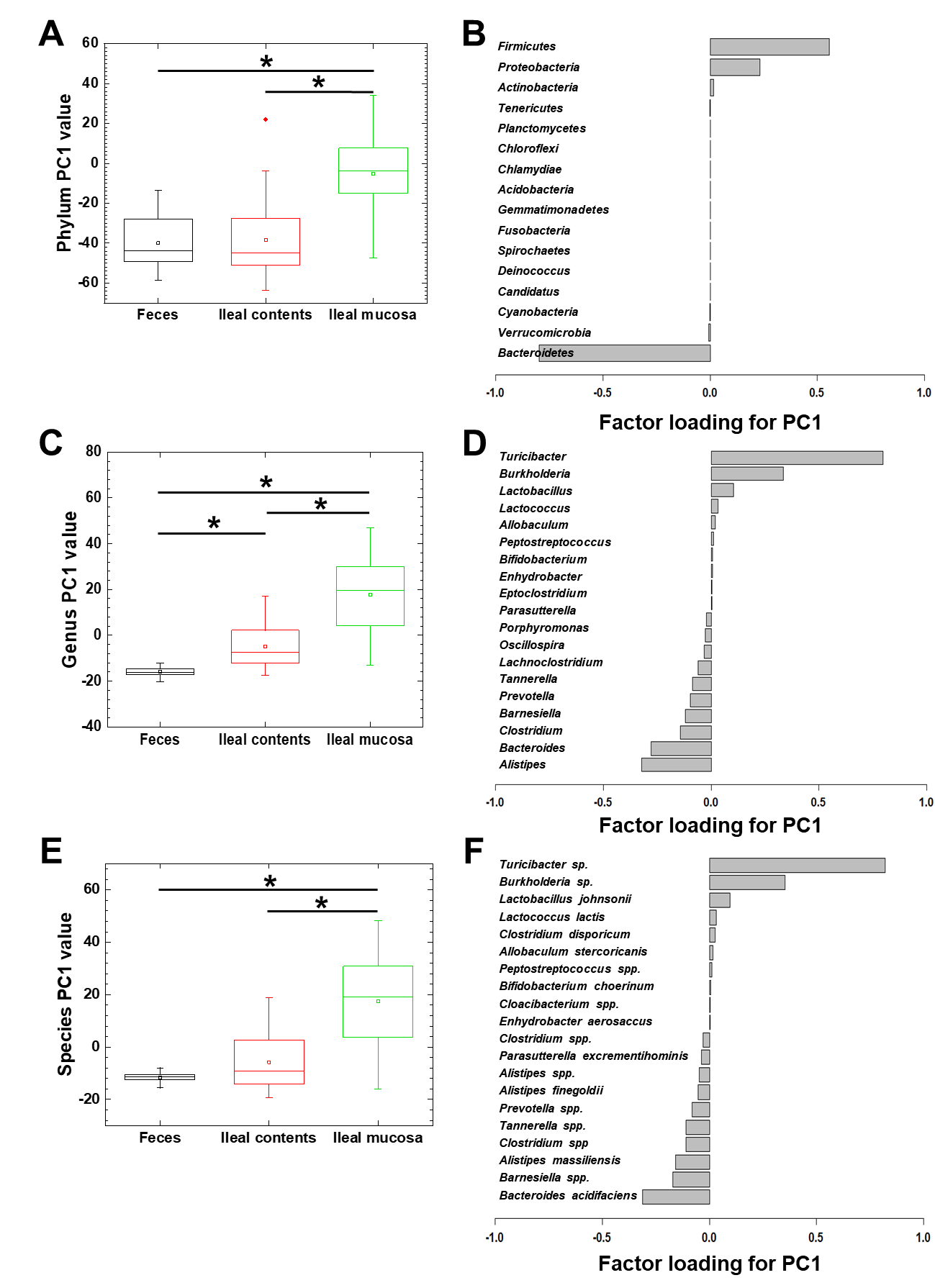


**Supplemental Figure 6.** We conducted PCA using microbiome data at the three taxonomical bacterial levels [phylum (**A, B**), genus (**C, D**), and species (**E, F**)] from the three anatomical sites (feces, ileal contents, and the ileal mucosa). We conducted PCA of microbiome data from feces (black), ileal contents (red), and the ileal mucosa (green). Proportion of variance for PC1 and PC2 accounts for 78% and 19% at the phylum level, 41% and 25% at the genus level, and 42% and 26% at the species level, respectively. PC1 values significantly different between feces versus the ileal mucosa, and ileal contents versus the ileal mucosa (**P* < 0.05) at the three taxonomical bacterial levels [phylum (**A**), genus (**C**), and species (**E**)]. At the phylum level, factor loading for PC1 showed that relative abundance of the phyla *Firmicutes* and *Bacteroidetes* positively and negatively correlated with PC1 values, respectively (**B**). At the genus level, the relative abundance of the species *Turicibacter.* most highly correlated with the PC1 values (**D**). At the species level, the relative abundance of the species *Turicibacter* sp*.* most highly correlated with PC1 values (**F**).

**
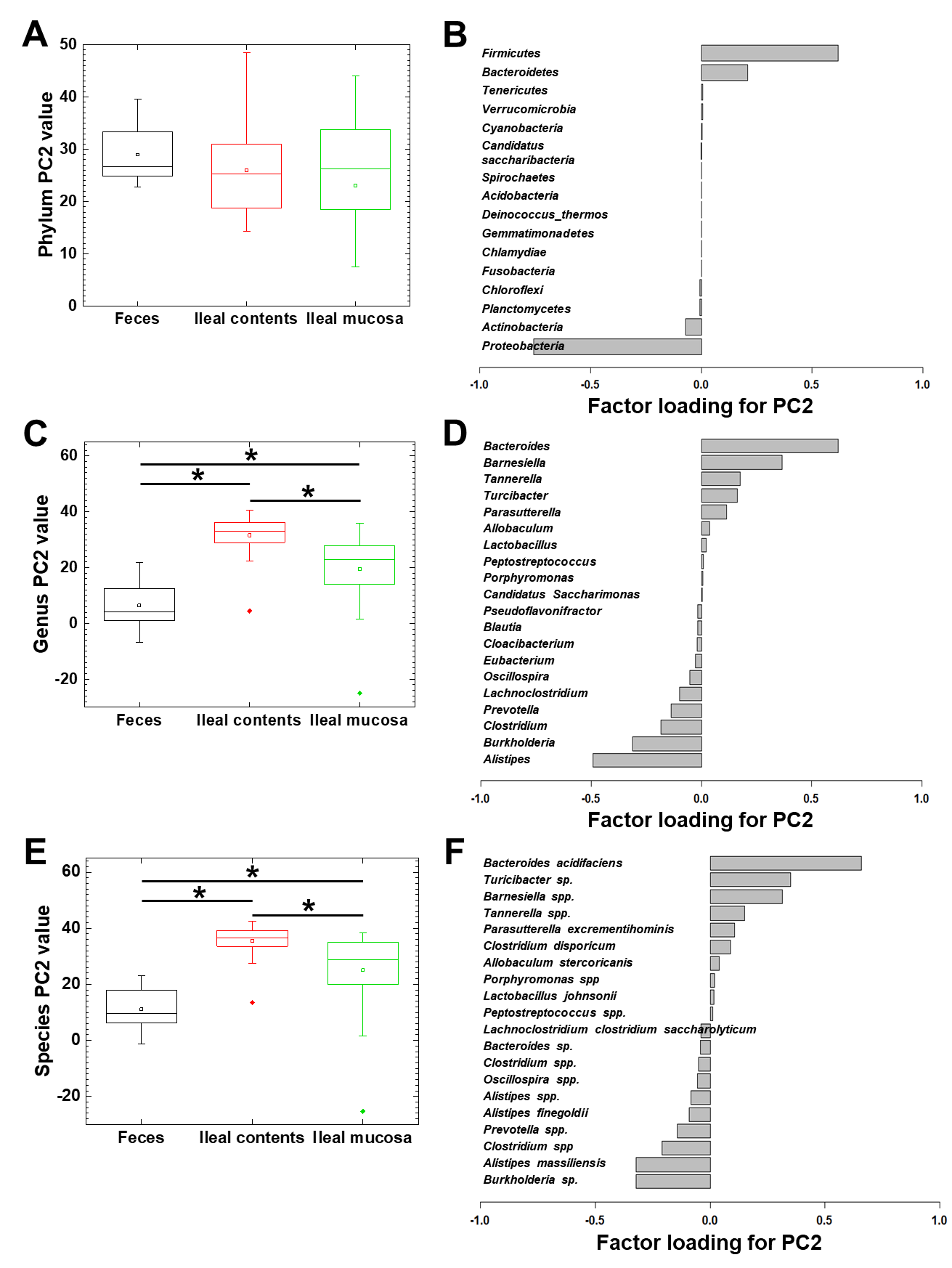
**

**Supplemental Figure 7.** PC2 values in PCA of microbiome data at the three taxonomical bacterial levels [phylum (**A, B**), genus (**C, D**), and species (**E, F**)] from the three anatomical sites (feces, ileal contents, and the ileal mucosa). We conducted PCA of microbiome data from feces (black), ileal contents (red), and the ileal mucosa (green). PC2 values were significantly different among all the three anatomical sites at the genus (**B**) and species (**C**) level, but not at the phylum level (**A**). Proportion of variance for PC2 accounts for 19% at the phylum level, 25% at the genus level and 26% at the species level. Factor loading for PC2 showed that the relative abundance of genera *Bacteroides* and *Alistipes* positively and negatively correlated with PC2 values, respectively (**D**). At the species level, the relative abundance of the species *Bacteroides acidifaciens* most highly correlated with PC2 values (**F**).


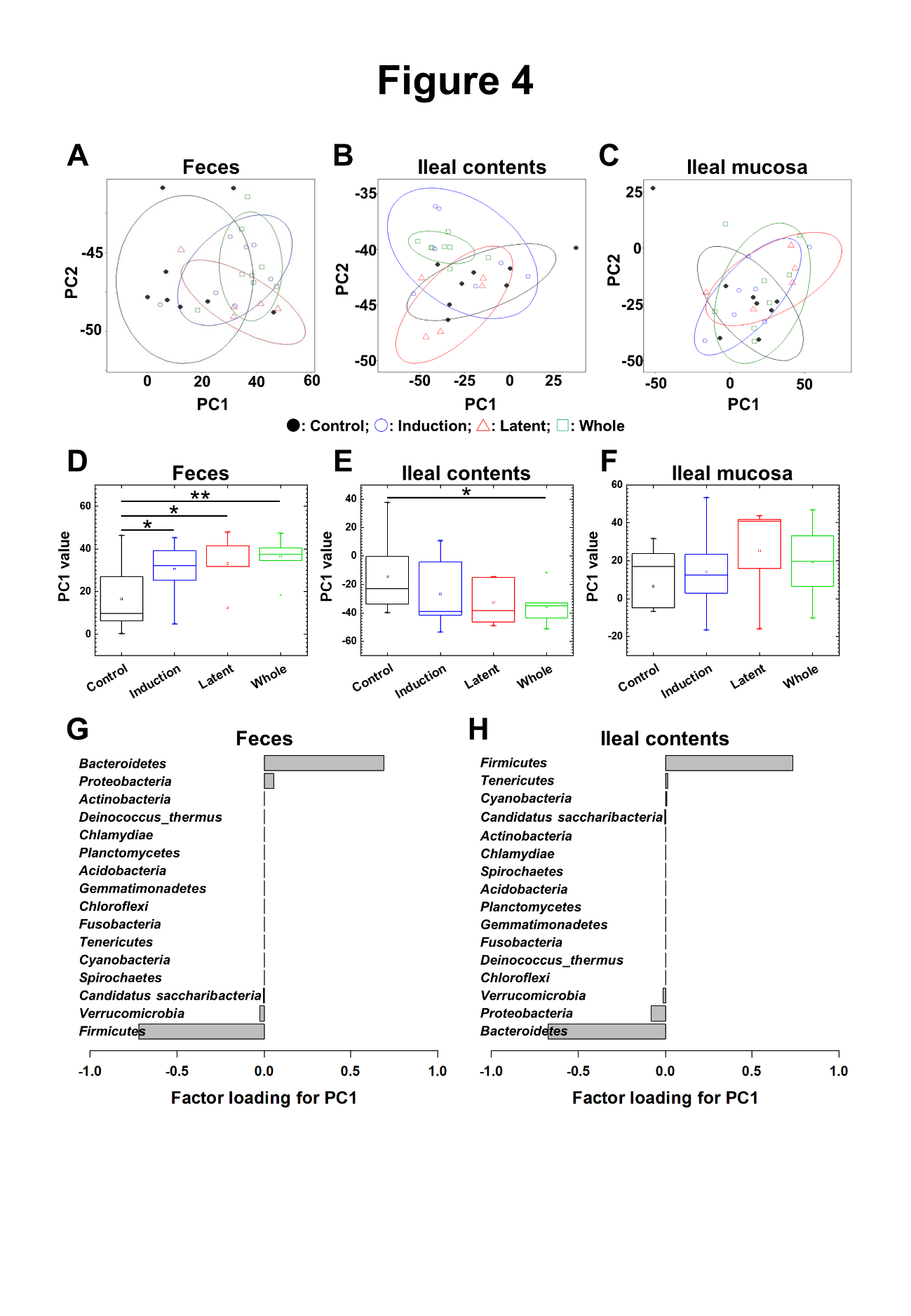


**Supplemental Figure 8.** PCA of microbiome data at the phylum level from the three CMG-treated (Induction, blue; Latent, red; and Whole, green) and the control (Control, black) groups. We conducted PCA using samples from the three anatomical sites: feces (**A**), ileal contents (**B**), and the ileal mucosa (**C**). Ellipses indicated an 80% confidence interval of each group. Proportion of variance of PC1 and PC2 were 96% and 2.7% in feces, 97% and 1.7% in ileal content, and 63% and 33% in the ileal mucosa. (**D**) In feces, PC1 values were significantly different between the Control versus Induction (**P* < 0.05), the Control versus Latent (**P* < 0.05), and the Control versus Whole groups (***P* < 0.01). (**G**) The phyla *Bacteroidetes* and *Firmicutes* correlated positively and negatively with PC1 values, respectively. (**E**) In ileal contents, PC1 values were significantly different between the Control versus Whole groups (**P* < 0.05). (**H**) The phyla *Firmicutes* and *Bacteroidetes* correlated positively and negatively with PC1 values, respectively. (**F**) In the ileal mucosa, PC1 values were not significantly different among the groups.


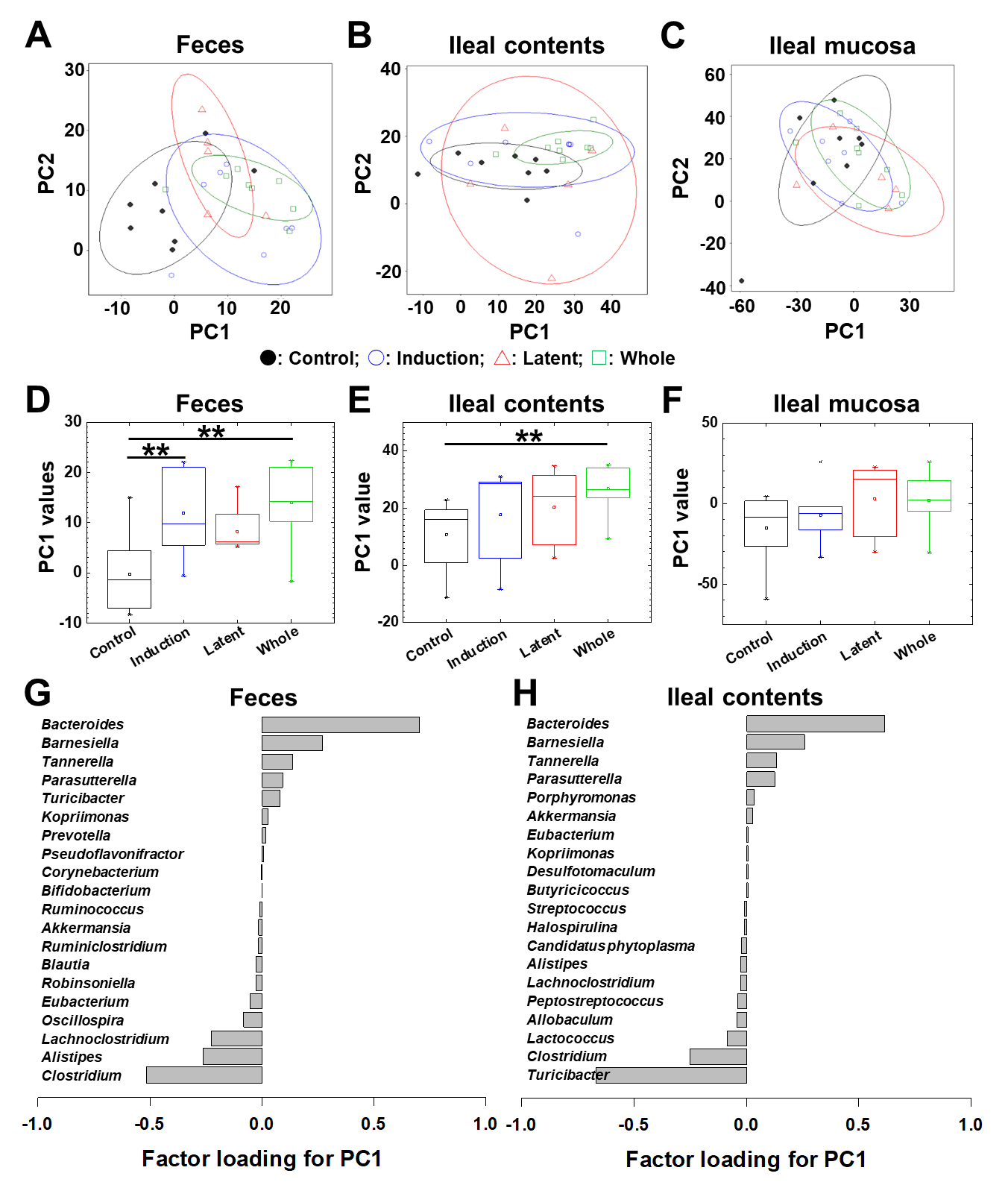


**Supplemental Figure 9.** PCA of microbiome data at the genus level from the three CMG-treated (Induction, blue; Latent, red; and Whole, green) and the control (Control, black) groups. We conducted PCA using samples from the three anatomical sites: feces (**A**), ileal contents (**B,**) and the ileal mucosa (**C**). Ellipses indicated an 80% confidence interval of each group. Proportion of variance of PC1 and PC2 were 47% and 23% in feces, 52% and 27% in ileal contents, and 43% and 36% in the ileal mucosa. In feces, PC1 values were significantly different between the Control versus Induction, and the Control versus Whole groups (***P* < 0.01) (**D**). The genera *Bacteroidetes* and *Clostridium* correlated positively and negatively with PC1 values, respectively (**G**). In ileal contents, PC1 values were significantly different between the Control versus Whole groups (***P* < 0.01) (**E**). The genera *Bacteroidetes* and *Turicibacter* correlated positively and negatively with PC1 values, respectively (**H**). In the ileal mucosa, neither PC1 values (**F**) nor PC2 values (data not shown) were statistically different among the four groups.

**
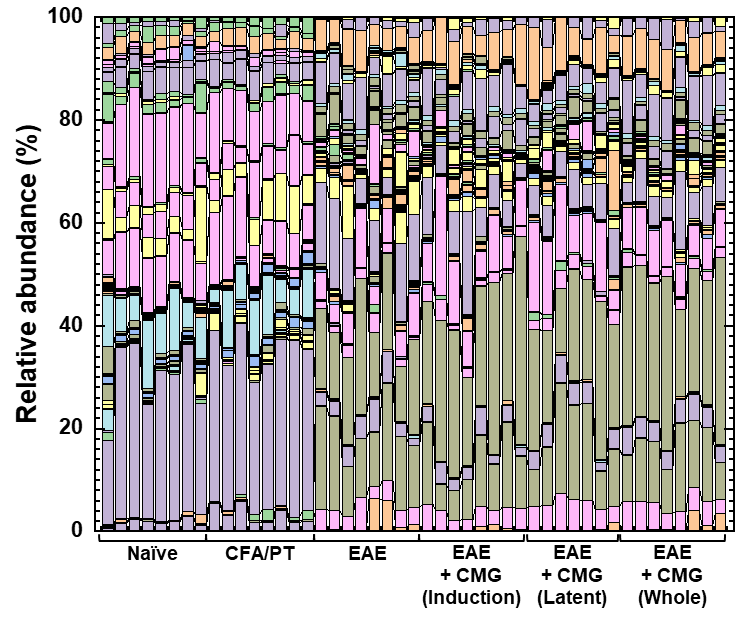
Supplemental Figure 10.** The relative abundance of fecal bacteria at the species level in control and EAE mice. Using 16S rRNA sequencing, we analyzed the relative abundance of individual bacteria from two age-matched control groups: naïve and complete Freund’s adjuvant (CFA)/pertussis toxin (PT)-injected mice. We found that the microbiota of the two control groups was similar. Then, we compared the microbiota from the controls with those from the four EAE groups: EAE mice with no treatment and three CMG-treated mice (Induction, Latent, and Whole). Compositional differences in the microbiota between the two control groups versus four EAE groups were much larger than the microbiota differences among the four EAE groups. Thus, in the following sections, we decided to evaluate the microbiota changes by comparing the three CMG-treated groups with the control EAE group, but not with the naïve or CFA/PT group.


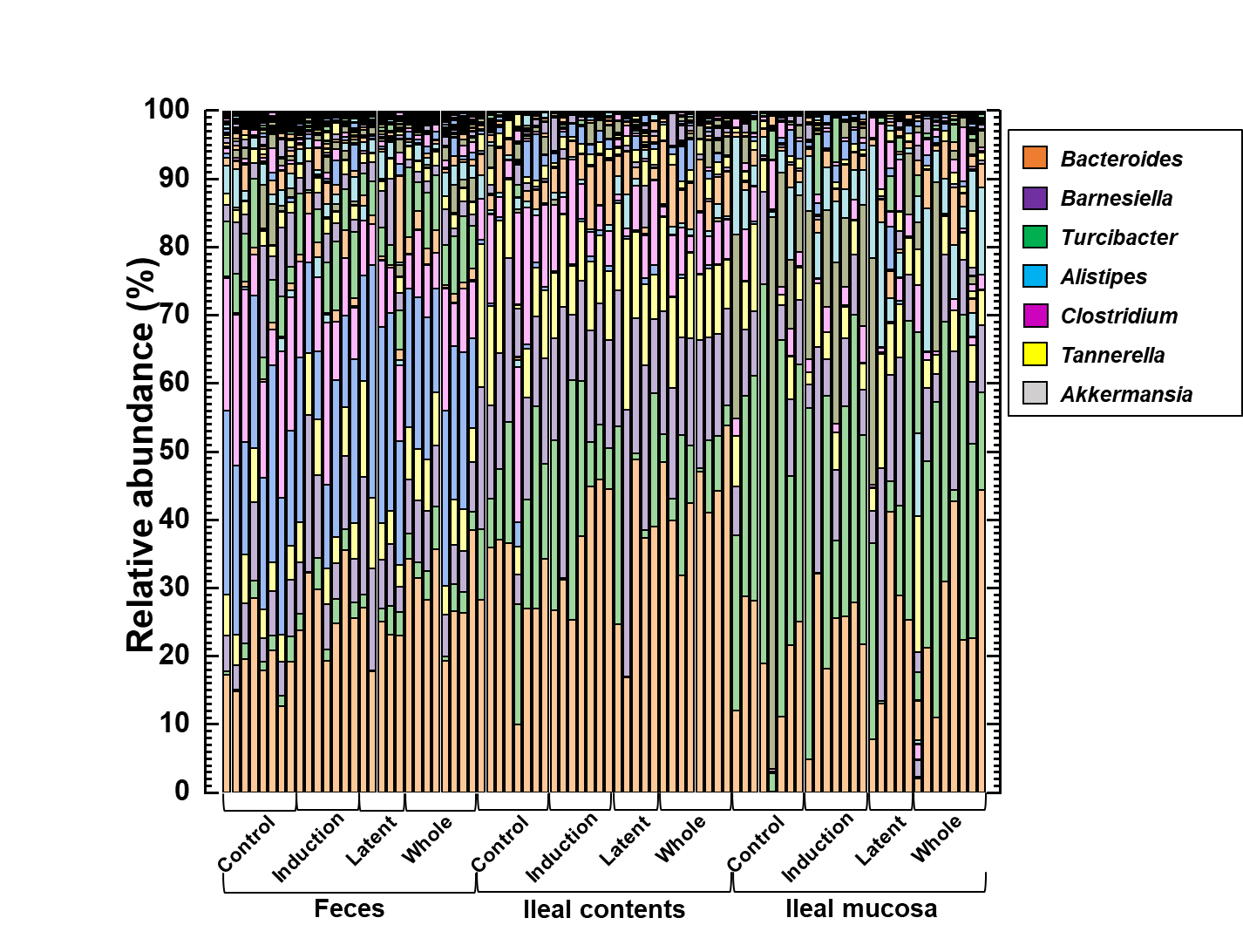
 **Supplemental Figure 11.** Relative abundance of bacteria from the three anatomical sites: feces, ileal contents, and the ileal mucosa. Using 16S rRNA sequencing, we analyzed the relative abundance of individual bacteria at the genus level. We harvested samples from the three CMG-treated (Induction, Latent, and Whole) and control (Control) groups. At the genus levels, compositional differences in the microbiota between the three anatomical sites were larger than the microbiota difference among the four groups. Sample numbers: Control, n = 8; Induction, n = 7; Latent, n = 5; and Whole, n = 8.

**
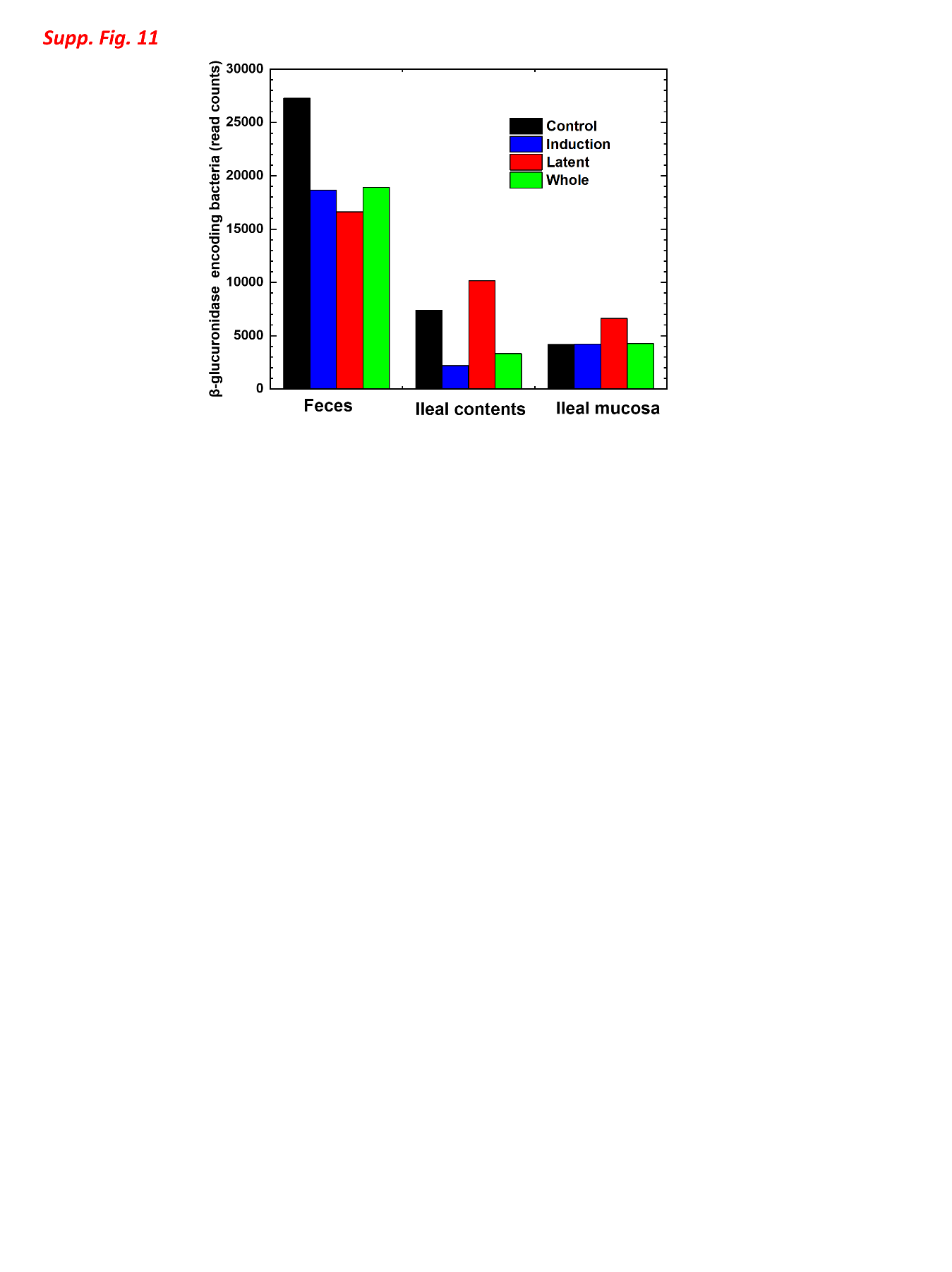
**

**Supplemental Figure 12.** Read counts of bacteria encoding β-glucuronidase in the three anatomical sites of the CMG-treated and control groups. Using PICRUSt, we found no significant differences in the read counts of bacteria encoding β-glucuronidase between the CMG-treated groups with the control group. We also compared the read counts of bacteria encoding β-glucuronidase among the three anatomical sites harvested from each group; we found read counts were significantly different between feces versus ileal contents, and feces versus the ileal mucosa in the Control (*P* < 0.001), in the Induction (*P* < 0.001), and in the Whole (*P* < 0.01) groups. *P* values were calculated by ANOVA.


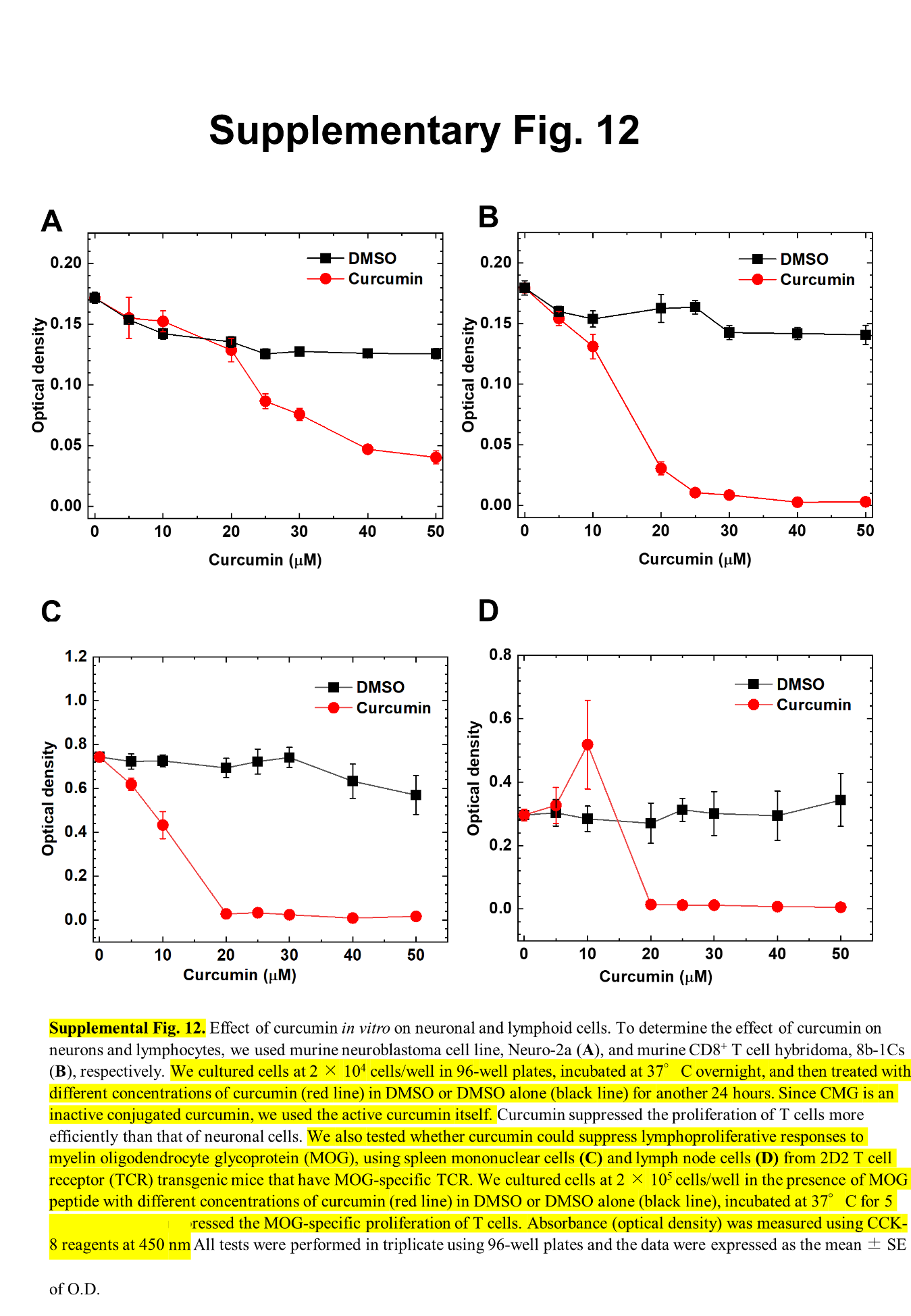


**Supplemental Figure 13.** Effects of curcumin *in vitro* on neuronal and lymphoid cells. To determine the effects of curcumin on neurons and lymphocytes, we used murine neuroblastoma cell line, Neuro-2a (**A**), and murine CD8^+^ T cell hybridoma, 8b-1Cs (**B**), respectively. We cultured cells at 2 × 10^4^ cells/well in 96-well plates, incubated at 37°C overnight, and then treated with different concentrations of curcumin (red line) in dimethyl sulfoxide (DMSO) or DMSO alone (black line) for another 24 hours. Since CMG is an inactive conjugated curcumin, we used the active curcumin itself. Curcumin suppressed the proliferation of T cells more efficiently than that of neuronal cells. We also tested whether curcumin could suppress the lymphoproliferative responses to MOG, using spleen mononuclear cells **(C)** and lymph node cells **(D)** from 2D2 T cell receptor (TCR) transgenic mice that have MOG-specific TCR. We cultured cells at 2 × 10^5^ cells/well in the presence of MOG peptide with different concentrations of curcumin (red line) in DMSO or DMSO alone (black line), incubated at 37°C for 5 days. Curcumin suppressed the MOG-specific proliferation of T cells. Absorbance (optical density, O.D.) was measured using CCK-8 reagents at 450 nm. All tests were performed in triplicate using 96-well plates and the data were expressed as the mean ± SE of O.D.


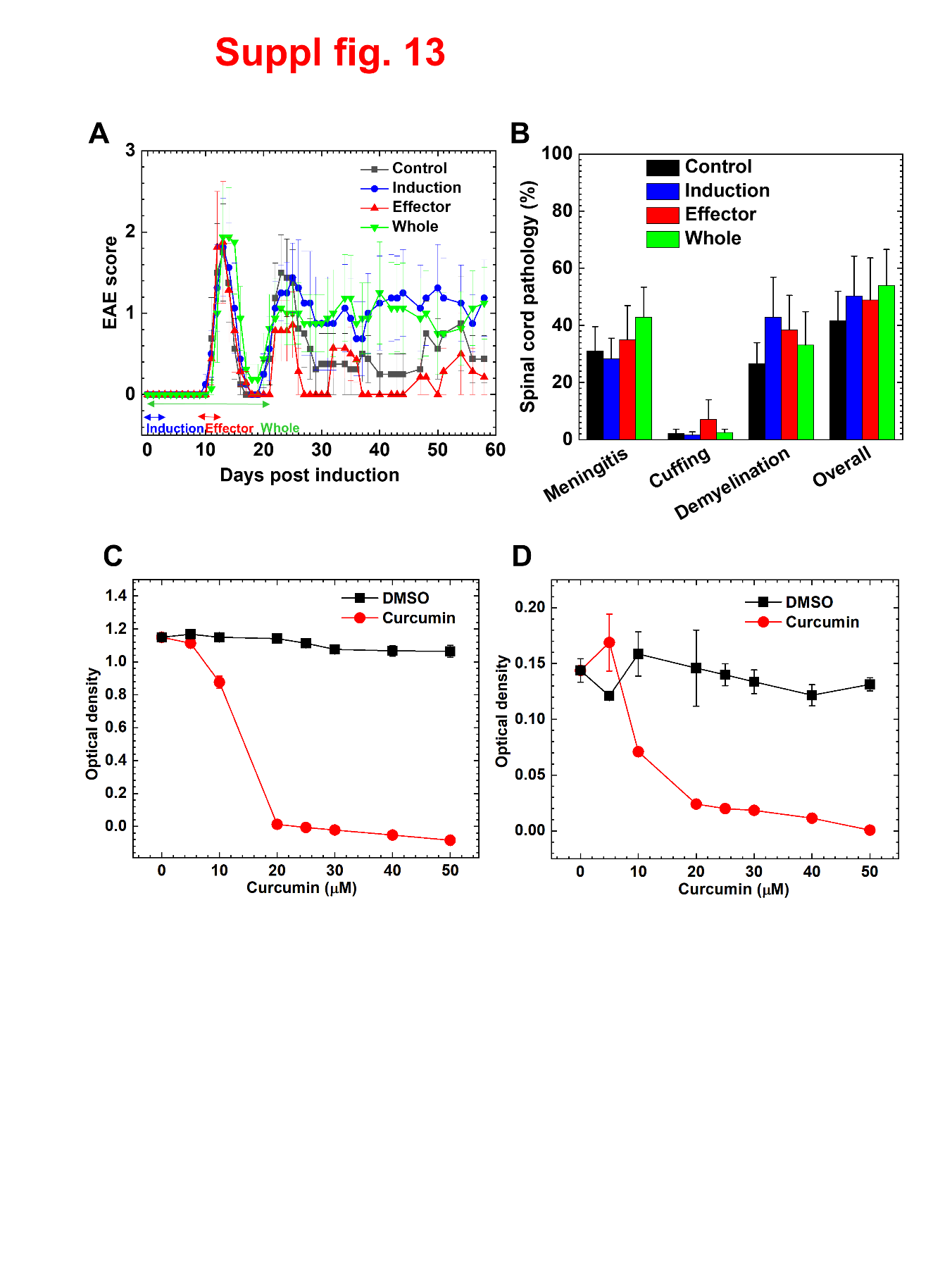


**Supplemental Figure 14.** CMG treatment for SJL/J mice with EAE induced by the myelin proteolipid protein (PLP)_139-151_ sensitization. For a relapsing-remitting EAE model, 4-week-old SJL/J mice (Charles River Laboratories Japan, Inc., Yokohama, Japan) were injected subcutaneously at the base of the tail with 148 μg (= 100 nmol) of the modiﬁed PLP_139-151_ peptide (VSLGKWLGHPDKF, United BioSystems, Herndon, VA) emulsified in CFA on day 0. We treated EAE mice with CMG daily on days 0–4 (Induction), on days 11–15 (Effector), or on days 0–21 (Whole). The control EAE mice (Control) were treated with PBS. Clinically, CMG treatment did not alter the clinical scores among the groups (**A**). We stained the spinal cord sections from EAE mice with Luxol fast blue to visualize the myelin. The spinal cord pathology scores of meningitis, perivascular inflammation (cuffing), demyelination, and overall pathology between the groups did not reach statistical differences (**B**). We tested whether curcumin could suppress the lymphoproliferative responses to PLP, using spleen mononuclear cells (**C**) and lymph node cells (**D**) from PLP-induced EAE mice. We cultured cells at 2 × 10^5^ cells/well in the presence of the PLP peptide with different concentrations of curcumin (red line) in DMSO or DMSO alone (black line), and incubated at 37°C for 5 days. Curcumin suppressed the PLP-specific proliferation of T cells dose-dependently. Absorbance (optical density, O.D.) was measured using the CCK-8 reagents at 450 nm. All tests were performed in triplicate using 96-well plates, and the data were expressed as the mean ± SE of O.D.

**Supplemental Table 1.** Effects of curcumin monoglucuronide (CMG) on clinical courses (**A**) and phenotypes of spinal cord infiltrates (**B**) of mice with myelin oligodendrocyte glycoprotein (MOG)_35-55_-induced experimental autoimmune encephalomyelitis (EAE)

1. Effects of CMG on clinical courses of EAE

| **Group (mouse number)** | **Cumulative score*** | **Maximum clinical score*** | **Onset of disease*** | **Incidence** | **Duration of disease* (Days)** |
| --- | --- | --- | --- | --- | --- |
| Control (n = 9) | 14.4 ± 4.3 | 2.8 ± 0.5 | 27.8 ± 1.6 | 89% (8/9) | 7.0 ± 1.7 |
| Induction (n = 8) | 9.4 ± 3.2 | 2.1 ± 0.5 | 29.4 ± 2.0 | 88% (7/8) | 5.8 ± 1.9 |
| Latent (n = 7) | 10.5 ± 4.1 | 1.8 ± 0.7 | 26.6 ± 2.0 | 71% (5/7) | 5.6 ± 1.5 |
| Whole (n = 8) | 6.1 ± 1.9  (*P* < 0.1) | 1.8 ± 0.5 | 27.8 ± 2.2 | 75% (6/8) | 4.5 ± 1.3 |

*Values are the mean ± standard error (SE)

**B.** Effects of CMG on the phenotypes of spinal cord infiltrates of EAE mice

| **Group** | **T cells**  **(%)^a^** | **B cells**  **(%)** | **Macrophages (%)** | **Neutrophils (number)^b^** | **Treg (number)** | **IgA^+^**  **cells** |
| --- | --- | --- | --- | --- | --- | --- |
| Control | 57.6 ± 10.7^c^ | 3.7 ± 0.6 | 7.4 ± 3.4 | 12.7 ± 4.9 | 53.2 ± 21.1 | ND^d^ |
| Induction | 52.3 ± 12.3 | 2.8 ± 0.9 | 11.7 ± 4.8 | 11.0 ± 3.0 | 73.1 ± 35.4 | ND |
| Latent | 46.3 ± 8.3 | 3.6 ± 0.8 | 8.5 ± 2.6 | 9.7 ± 3.52 | 49.2 ± 17.9 | ND |
| Whole | 32.7 ± 12.4 | 4.2 ± 0.8 | 10.5 ± 2.4 | 10.3 ± 3.1 | 50.6 ± 21.1 | ND |

Markers used: T cells, CD3; B cells, B220; macrophages, F4/80; neutrophils, Ly-6G, regulatory T (Treg) cells, Foxp3; and IgA-producing B cells, IgA.

^a^, %: number of cells positive for each immune cell marker / total cell number in perivascular infiltrates x 100

^b^, number: number of cells positive for each immune cell per spinal cord quadrant

^c^, Values are the mean ± standard error (SE). Three to four mice per group

^d^, ND: IgA-producing cells were not detected

**Supplemental Table 2**. Compositional differences of microbiota between the three anatomical sites^a^

| **Taxa** | **Number of significantly different taxa** | | | **bacteria no./ total taxon no.**^b^ |
| --- | --- | --- | --- | --- |
|  | **Feces versus Ileal contents** | **Feces versus Ileal mucosa** | **Ileal contents versus**  **ileal mucosa** |  |
| **Phylum**^c^ | Total = 3  Feces> Ileal contents:1  Feces< Ileal contents:2 | Total = 5  Feces > Ileal mucosa:3  Feces < Ileal mucosa:2 | Total = 6  Ileal contents > mucosa:3  Ileal contents < mucosa:3 | 7/16 |
| **Genus**^d^ | Total = 53  Feces> Ileal contents:38  Feces< Ileal contents:15 | Total = 70  Feces > Ileal mucosa:52  Feces < Ileal mucosa:18 | Total = 42  Ileal contents > mucosa:32  Ileal contents < mucosa:10 | 78/252 |
| **Species**^d^ | Total = 129  Feces> Ileal contents:101  Feces< Ileal contents:28 | Total = 140  Feces > Ileal mucosa:115  Feces < Ileal mucosa:25 | Total = 72  Ileal contents > mucosa:58  Ileal contents < mucosa:14 | 160/387 |

^a^Compositional differences were compared, using data of total 28 samples (Control, n = 8; Induction, n = 7; Latent, n = 5; and Whole, n = 8) per each anatomical site.

^b^Total number of bacterial taxa whose relative abundance were significantly different between feces, ileal contents, and the ileal mucosa by ANOVA with Fisher’s PLSD test (*P* < 0.05).

^c^At the phylum level, we found that relative abundance of three, five and six phyla significantly differed in feces versus ileal contents, feces versus the ileal mucosa, and ileal contents versus the ileal mucosa, respectively. For example, the ileal mucosal samples had statistically lower abundance of *Bacteroidetes* and higher abundance of *Firmicutes* and *Proteobacteria* than the fecal and ileal content samples [mean relative abundance ± SE: *Bacteroidetes*, 69.3 ± 1.9 (feces), 67.4 ± 2.7 (ileal contents), 40.2 ± 3.5 (ileal mucosa); *Firmicutes*, 26.8 ± 2.0 (feces), 25.7 ± 2.9 (ileal contents), 42.5 ± 2.7 (ileal mucosa); *Proteobacteria*, 2.6 ± 0.3 (feces), 5.1 ± 0.5 (ileal contents), 15.0 ± 3.1 (ileal mucosa); *P* < 0.05, ANOVA].

^d^The relative abundance of individual bacterial genera and species were significantly different between the three anatomical sites. The numbers of significantly different relative abundance at the genus and species levels were as follows: feces versus ileal contents (genera, 53; species, 129), feces versus the ileal mucosa (genera, 70; species, 140), and ileal contents versus the ileal mucosa (genus, 42; species, 72). These compositional differences in the microbiota among the three anatomical sites were larger than the microbiota differences among the CMG-treated and Control groups (Tables 2–4).

| **Group (mouse number)** | **Symptomatic mouse number (Incidence %)** | **Mean number of relapse (**± **SE)** |
| --- | --- | --- |
| Control (8) | 6 (75.0 %) | 2.7 ± 0.3 |
| Induction (8) | 6 (75.0 %) | 3.0 ± 0.5 |
| Effector (8) | 5* (62.5 %) one mouse died^†^ | 2.2 ± 0.5 |
| Whole (8) | 6 (75.0 %) | 2.7 ± 0.4 |
| Total (32) | 23 (71.8 %) |  |

**Supplemental Table 5.** Effects of CMG on the incidence of EAE in the mice sensitized with myelin proteolipid protein (PLP)_139-151_

*Total mice developed EAE; †one out of five symptomatic mice scored 5 (moribund)
